# Green Synthesis, Characterization, and the Prediction of Its Thermochemical Properties of the Isomers 3,4- and 3,5-Bis(1,3-dihydro-1,3-dioxo-2H-isoindol-2-yl)benzoic Acid

**DOI:** 10.1021/acsomega.6c04687

**Published:** 2026-07-30

**Authors:** Diego Espinosa-Morales, Fausto Díaz-Sánchez, María J. Sáenz de Miera-Ramos, Miguel A. García-Castro, Luis H. Gutiérrez-Gámez, Francisco Paraguay-Delgado

**Affiliations:** † 657578 Facultad de Ingeniería Química de la Benemérita Universidad Autónoma de Puebla, 18 Sur y Av. San Claudio, Puebla, Puebla C.P. 72570, México; ‡ Centro de Investigación en Materiales Avanzados SC, Miguel de Cervantes 120, Complejo Industrial Chihuahua, Chihuahua, Chihuahua C.P. 31136, México

## Abstract

The solid-state synthesis of the 3,4- and 3,5-bis­(1,3-dihydro-1,3-dioxo-2H-isoindole-2-yl) benzoic acid isomers (hereafter referred to as DIAs) is reported using a solvent-free strategy aligned with the principles of green chemistry. A previously reported methodology was employed, and the relationship between molecular structure and thermal behavior was evaluated. Structural characterization was performed by infrared spectroscopy (FTIR), proton nuclear magnetic resonance (^1^H NMR), and mass spectrometry, confirming the formation of the proposed bisphthalimide derivatives. Thermal properties were investigated by differential scanning calorimetry (DSC) and thermogravimetric analysis (TGA), allowing the determination of thermal stability, melting points, and associated thermal events. Additionally, theoretical quantum calculations were employed to predict the thermochemical properties of the DIAs, as well as those of 3,4- and 3,5-diaminobenzoic acid, which are useful for calculating the reaction equilibrium properties. The combined experimental and computational approach provides more in-depth insight into the physicochemical behavior of these systems and demonstrates the feasibility of the proposed solvent-free synthetic strategy.

## Graphical abstract

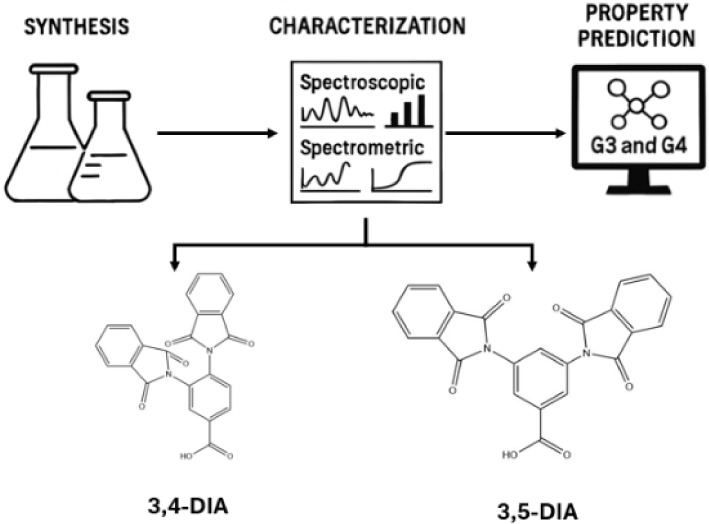

## 1 Introduction

Synthesis has been a fundamental part of scientific research^1^ since new molecules and functional materials with specific characteristics have been obtained that have applications in different fields and that solve problems.^2,3^ However, there is an interest in green synthesis routes where the use of solvents is avoided and the products are molecules that do not pollute the environment.^4–6^ As a result of this, interest in imide compounds, in particular phthalimides and their derivatives, increased due to their high thermal stability and electronic distribution,^7,8^ in addition to the fact that applications have been discovered in areas such as medicine,^4,9^ materials science,^10,11^ and chemical catalysis.^12–14^

Within this group, aromatic biphthalimides and diimides have attracted even greater interest as they have an electronic distribution that impacts their physicochemical behavior, as well as a greater possibility of functionalization.^15,16^ The obtaining of these compounds usually involves condensation reactions between phthalic anhydrides and aromatic or aliphatic diamines,^18,19^ and architecture is controlled through the selection of precursors. In addition, its simplicity of synthesis paths, high yields, and reproducibility are favored.^17^ However, variations in structure can generate significant changes in spectroscopic, thermal, and electronic behavior, so characterization is essential.^18^

The characterization of the compounds can be integrated into spectroscopic techniques such as FTIR and NMR,^19,20^ in which the traditional bands of the imide group are observed as well as the integrity of the aromatic structure. In addition, thermal techniques such as DSC and TGA can be applied, in which physical transitions and information on their thermal stability are observed.^21–23^ In a complementary way, spectrometric techniques can be applied, such as mass spectrometry, which provides information on the structure of the synthesized molecule, and optical techniques such as UV–vis, which can provide indications about electronic transitions, specifically compounds with potential application in functional materials.^24^

Recently, computational methods, such as the G3 and G4 ^25^ composite methods, have been increasingly employed,^26^ as they have become well-established tools for predicting thermodynamic and energetic properties, as well as the spectra of organic molecules.^27,28^ The integration of theoretical methods with experimental results enables a more in-depth understanding of molecular stability and the influence of structure on the observed properties.^29^

Therefore, in the present work, we focus on the solvent-free synthesis of the structural isomers 3,4- and 3,5-bis­(1,3-dihydro-1,3-dioxo-2H-isoindole-2-yl)­benzoic acid (hereafter referred to as DIAs, Figure 1). These compounds have previously been prepared through solution-based methodologies involving the formation of poly­(amic acid) intermediates followed by chemical imidization using dehydrating and cyclization agents, such as acetic anhydride and tertiary amines, or by high-temperature syntheses conducted in the presence of organic solvents. Reported reaction times range from 0.5 to 14 h, with yields varying between 57 and 90%.^30–33^

**Figure 1:**
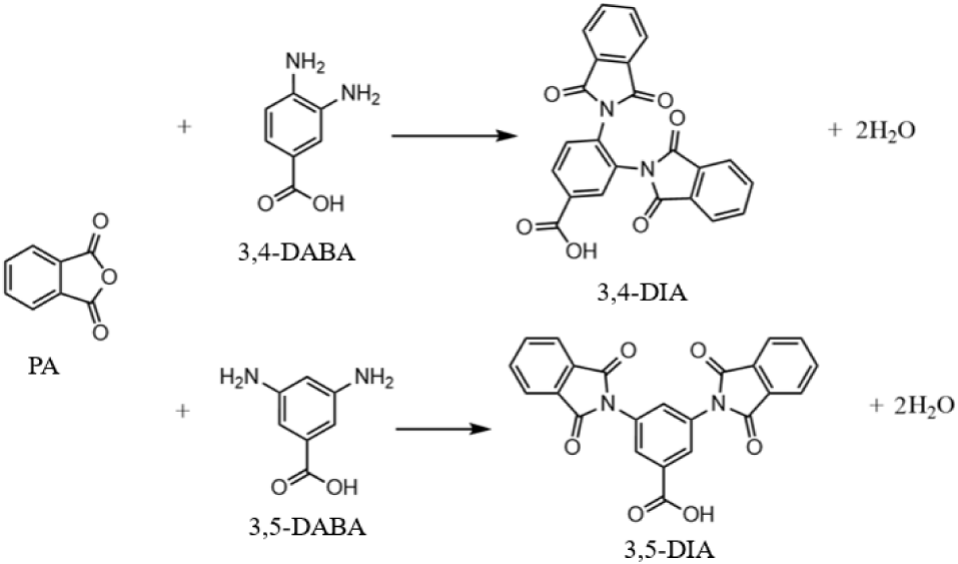
Synthesis reaction of DIAs.

Previous studies have mainly focused on synthetic procedures and structural characterization by infrared spectroscopy (IR), proton nuclear magnetic resonance spectroscopy (^1^H NMR), and, in some cases, thermogravimetric analysis (TGA).^30–33^ However, despite the growing interest in aromatic imide derivatives due to their remarkable thermal stability and potential applications as functional materials, a detailed evaluation of their thermodynamic and thermochemical properties has not yet been reported.

In this context, the present contribution not only provides an environmentally friendly, solvent-free synthetic approach to both DIA isomers but also presents a comprehensive physicochemical characterization combining spectroscopic, spectrometric, and thermal techniques. Furthermore, the experimental results are complemented by quantum chemical calculations, allowing the determination and rationalization of key thermochemical parameters and the energetic differences associated with positional isomerism. This integrated experimental-theoretical approach provides new insights into the structure–stability relationships of these aromatic diimides and contributes valuable information that is currently absent from the literature.

## 2 Experimental Procedure

### 2.1 Materials and Instruments

Phthalic anhydride [CAS 85-44-9], 3,5-diaminobenzoic acid [CAS 535-87-5], and 3,4-diaminobenzoic acid [CAS 619-05-6], hereafter referred to as PA, 3,5-DABA, and 3,4-DABA, respectively, were purchased from Sigma-Aldrich with mass fractions of 99%, 98%, and 97%. The reagents were used as received, without further purification.

Fourier-transform infrared (FTIR) spectroscopy was performed using a PerkinElmer spectrometer with a resolution of 0.5 cm^–1^. Thermogravimetric analysis was conducted using a TGA Jupiter NETZSCH STA 449 F3 system, while the melting point was determined by differential scanning calorimetry (DSC) using a Mettler Toledo DSC-1 instrument. Proton nuclear magnetic resonance ^1^H NMR) spectroscopy was carried out on a 500 MHz Bruker NMR spectrometer, using DMSO-*d*_6_ as the solvent. Finally, mass spectrometry was performed using a JEOL MStation JMS-700 system.

### 2.2 Synthesis of 3,4-DIA and 3,5-DIA

3,4-Bis­(1,3-dihydro-1,3-dioxo-2H-isoindol-2-yl)­benzoic acid and 3,5-bis­(1,3-dihydro-1,3-dioxo-2H-isoindol-2-yl)­benzoic acid, referred to as 3,4-DIA and 3,5-DIA by our research group, respectively, were synthesized following the methodology proposed by Díaz et al.,^34^ through solid-state synthesis using 0.7256 g of phthalic anhydride (referred to as PA) and 3,N-DABA (0.3765 g). As in the approach described by Díaz et al.^34^ variations in the order of reagent addition were tested, considering their respective melting points. The most efficient synthetic route involved heating PA to its melting point (403.95 K) and stirring vigorously until a homogeneous mixture was obtained. 3,N-DABA was then added, and the temperature was increased to its melting point (501.15 K), maintaining constant agitation to ensure complete fusion of 3,N-DABA. The reagents were added in this order, from lowest to highest melting point, with the aim of preventing their decomposition and ensuring the lowest possible amount of trace impurities in the final product.

After cooling, the reaction mixture was dried under an absolute pressure of 26 cmHg at an average temperature of 523.15 K for 3 h. The resulting product was a green solid (yield of 89% for the 3,4-DIA) and a white solid (yield of 85% for the 3,5-DIA). Characterization of 3,4- and 3,5-DIA was performed by IR spectroscopy, ^1^H NMR spectroscopy, and mass spectrometry (using electron ionization (EI) at 70 eV).

In the case of the 3,4-DIA **IR** spectrum (cm^–1^): 2881 (*ν*O–H), 1789–1759 (*ν*C=O), 1435–1278 (*ν*C–N and *ν*C–O imide groups), 893–715 (*ν*C–O, aromatic ring). ^**1**^ **H NMR** spectrum (500 MHz, DMSO-*d*_6_) δ ppm: 12.95 (1H, s, H^1^), 8.17 (dd, 1.6 Hz, 2H), 7.97–7.86 (m, 4H), 7.84–7.73 (m, 4H), 7.63 (d, J = 8.5 Hz, 1H). **MS** (70 eV, *m*/*z*, rel. int.): 412 (M^+^), 264 (C_18_H_4_N_2_O_1_^+^), 247 (C_18_H_3_N_2_^+^), 199 (C_14_H_3_N_2_^+^), and 50 (C_4_H_2_^+^). And the 3,5-DIA **IR spectrum (cm** ^**–1**^ **)**: 2850 (*ν*–OH, carboxylic acid), 1720 (*ν*C=O, carboxylic acid), 1600 (*ν*C=O, imide), 1370 (*ν*C–O, carboxylic acid), 1300–1100 (*ν*C–N–C), 850–700 (*ν*C–H, central aromatic ring). ^**1**^ **H NMR** spectrum (500 MHz, DMSO-*d*_6_) δ ppm: 8.15 (d, J = 1.9 Hz, 2H), 8.01 (dd, J = 5.5, 3.1 Hz, 4H), 7.93 (dd, J = 5.5, 3.1 Hz, 4H), 7.87 (s, 1H). **MS** (E.I 70 eV, *m*/*z*, rel. int.): 412 (M^+^), 282 (C_18_H_6_N_2_O_2_^+^), 152 (C_11_H_6_N^+^), 76 (C_6_H_4_^+^), 50 (C_4_H_2_^+^).

The melting temperatures of 3,4-DIA and 3,5-DIA were determined using a Mettler Toledo DSC1 Star system, previously calibrated for temperature and heat flow using indium. The heating rate was set to 3 K·min^–1^ with a nitrogen flow rate of 80 cm^3^·min^–1^, no other thermal transition was observed during heating from 298.15 to 653.15 K. The fusion temperature of each DIA was defined as the onset temperature of the melting endotherm obtained from the DSC thermogram, since melting is immediately followed by thermal decomposition.

The mass loss due to thermal decomposition was determined using a TGA Jupiter NETZSCH STA 449 system, with a heating rate of 50 K·min^–1^ and a nitrogen flow rate of 80 cm^3^·min^–1^ up to 1073.15 K.

## 3 Computational Procedure

Quantum chemical calculations were performed using the Gaussian16 software package. Initially, a conformational search was conducted for both the reactants (3,4- and 3,5-DABA) and the products (3,4- and 3,5-DIA). The identified conformers were optimized using Density Functional Theory (DFT) with the B3LYP functional and the 6-311++G­(d,p) basis set.^35^ Harmonic vibrational frequency calculations were performed at this same level of theory to ensure the absence of imaginary frequencies. For each compound, the structure with the lowest energy was selected as the most stable conformer to proceed with the rigorous thermochemical evaluation.

To obtain highly accurate thermochemical properties, standard composite ab initio methods were employed on the selected stable conformers. For the diaminobenzoic acids (DABA), the G3, G4, and CBS-QB3 ^36^ levels of theory were used. CBS-QB3 reduces computational cost compared to G4 while maintaining good accuracy. For the highly functionalized DIA products, the G3­(MP2) methodology was applied due to the exponential increase in computational cost associated with the larger number of electrons (212 e^–^ vs 80 e^–^ in DABA).

It is important to emphasize that the final thermochemical data were extracted directly from the composite methods’ outputs to maintain strict thermodynamic consistency. These methods internally execute their own standardized sequence of geometry optimization and frequency calculations, automatically applying specific inherent scaling factors for zero-point energy (ZPE) and thermal corrections. The implicit frequency scaling factors utilized by these methods are 0.8929 for G3 and G3­(MP2) (evaluated at the HF/6-31G­(d) level), 0.9854 for G4 (evaluated at the B3LYP/6-31G­(2df,p) level), and 0.9900 for CBS-QB3 (evaluated at the B3LYP/CBSB7 level). Absolute entropies, standard enthalpies, and standard Gibbs free energies of formation at 298.15 K were directly taken from these composite method outputs, avoiding any hybrid thermochemical treatment. Finally, the standard enthalpies of formation in the gas phase were calculated using atomization reactions (eqs 1 and 2).

1 $$C_{7}H_{8}N_{2}O_{2}\left(g\right)\to \mathrm{7 C}\left(g\right)+\mathrm{8 H}\left(g\right)+\mathrm{2 O}\left(g\right)+\mathrm{2 N}\left(g\right)$$

2 $$C_{23}H_{12}N_{2}O_{6}\left(g\right)\to \mathrm{23 C}\left(g\right)+\mathrm{12 H}\left(g\right)+\mathrm{6 O}\left(g\right)+\mathrm{2 N}\left(g\right)$$

## 4 Results and Discussion

### 4.1 Synthesis and Characterization

The solid-state synthesis method proposed by Díaz et al. for the preparation of acid biphthalimides allows precise control over critical parameters such as temperature and reaction time.^34^ Furthermore, by avoiding the use of solvents that may leave residual traces or undesirable impurities, the final product typically exhibits considerably higher purity compared to those obtained through alternative synthetic methods.

Three different synthesis routes were evaluated, according to the order in which each reagent was added: 1) 3,N-DABA was melted first, followed by PA addition; 2) PA was melted first, followed by 3,N-DABA addition; 3) 3,N-DABA and PA were pulverized, mixed, and melted together.

The following results were obtained: 1) when 3,N-DABA was melted first and subsequently PA was added, the mixture underwent decomposition, turning dark; 2) when PA was melted first and 3,N-DABA was then added, a homogeneous and uniform mixture was obtained, exhibiting a light gray color; 3) when both reactants were added simultaneously, clumps were formed that were difficult to break apart even under constant stirring, leading to incomplete reaction of the reagents and the formation of impurities.

The reaction time for the second route was 0.5 h. The obtained products were purified by sublimation at 523.15 K under a vacuum pressure of 500 mmHg. After this treatment, the substance reached a yield of 89% in the case of the 3,4-DIA and 85% for the 3,5-DIA. Compared to previously reported methodologies for the synthesis of related phthalimide derivatives using glacial acetic acid as the reaction medium, the current solvent-free protocol provided comparable yields, values like those reported by Kuznetsov et al.^37^ In addition, the reaction time was significantly reduced from 1.5 to 0.5 h. Furthermore, no condensing agents or auxiliary reagents were required, and water was generated as the only byproduct. In contrast, conventional synthetic approaches often rely on organic solvents and dehydrating agents such as acetic anhydride or other activating reagents, which increase waste generation and environmental load.^38^ These features together highlight the advantages of the proposed methodology from both practical and green chemistry perspectives, in line with the principles of green chemistry that emphasize waste minimization and the use of safer reagents.^39^

The purified 3,4-DIA and 3,5-DIA were characterized by FTIR, ^1^H NMR, MS, DSC, and TGA.

The FTIR spectrum of 3,4-DIA revealed the characteristic absorption bands associated with the proposed bisphthalimide structure. The intense bands observed at 1789–1759 cm^–1^ were assigned to the stretching vibrations of the imide carbonyl groups (ν C=O), while the absorptions in the 1435–1278 cm^–1^ region corresponded to the C–N and C–O stretching modes of the imide ring. In addition, bands located between 893 and 715 cm^–1^ were attributed to aromatic ring vibrations, whereas the absorption at 2881 cm^–1^ was assigned to the O–H stretching vibration of the carboxylic acid group. These signals are consistent with the formation of the target 3,4-DIA structure.

The ^1^H NMR spectrum of 3,4-DIA exhibited five signals located at δ 12.95, 8.17, 7.97–7.87, 7.85–7.74, and 7.63 ppm. The signal at δ 12.95 ppm was assigned to the carboxylic acid proton, while the remaining resonances corresponded to the aromatic protons of the benzoic acid and phthalimide moieties. The observed chemical shifts and signal distribution are in agreement with the proposed structure of the 3,4-isomer. Furthermore, the mass spectrum showed the molecular ion peak at *m*/*z* 412 together with fragment ions at *m*/*z* 264, 247, 199, and 50, supporting the molecular structure of the synthesized compound.

The FTIR spectrum of 3,5-DIA also displayed the characteristic absorptions of both imide and carboxylic acid functionalities. The absorption bands observed in the 1300–1100 cm^–1^ region were assigned to the C–N–C stretching vibrations of the imide group, whereas the bands at 1720 and 1600 cm^–1^ corresponded to carbonyl stretching vibrations. Additionally, the absorption at 1370 cm^–1^ was attributed to the C–O stretching mode of the carboxylic acid group, while the broad band at 2850 cm^–1^ was associated with the O–H stretching vibration. These results are consistent with the successful formation of the 3,5-DIA isomer.

The ^1^H NMR spectrum of 3,5-DIA showed four signals at δ 8.154, 8.01, 7.93, and 7.876 ppm, corresponding to the aromatic proton environments expected for the proposed structure. The lower number of resonances compared with the 3,4-isomer is consistent with the higher symmetry of the 3,5-DIA molecule. In addition, the mass spectrum exhibited the molecular ion peak at *m*/*z* 412 and characteristic fragment ions at *m*/*z* 282, 152, 104, 76, and 50, further supporting the identity of the synthesized compound. The IR spectrum, the signals obtained by ^1^H NMR, the mass spectrum, and the fragmentation path are shown in the Supporting Information Figures S1–S8.

For the thermal characterization of the DIAs by DSC, the experiments were performed using sealed aluminum crucibles. The resulting thermograms are shown in Figure 2, where endothermic peaks associated with melting can be observed. The onset temperatures of the melting endotherms were 544.36 and 682.29 K for 3,4-DIA and 3,5-DIA, respectively.

**Figure 2:**
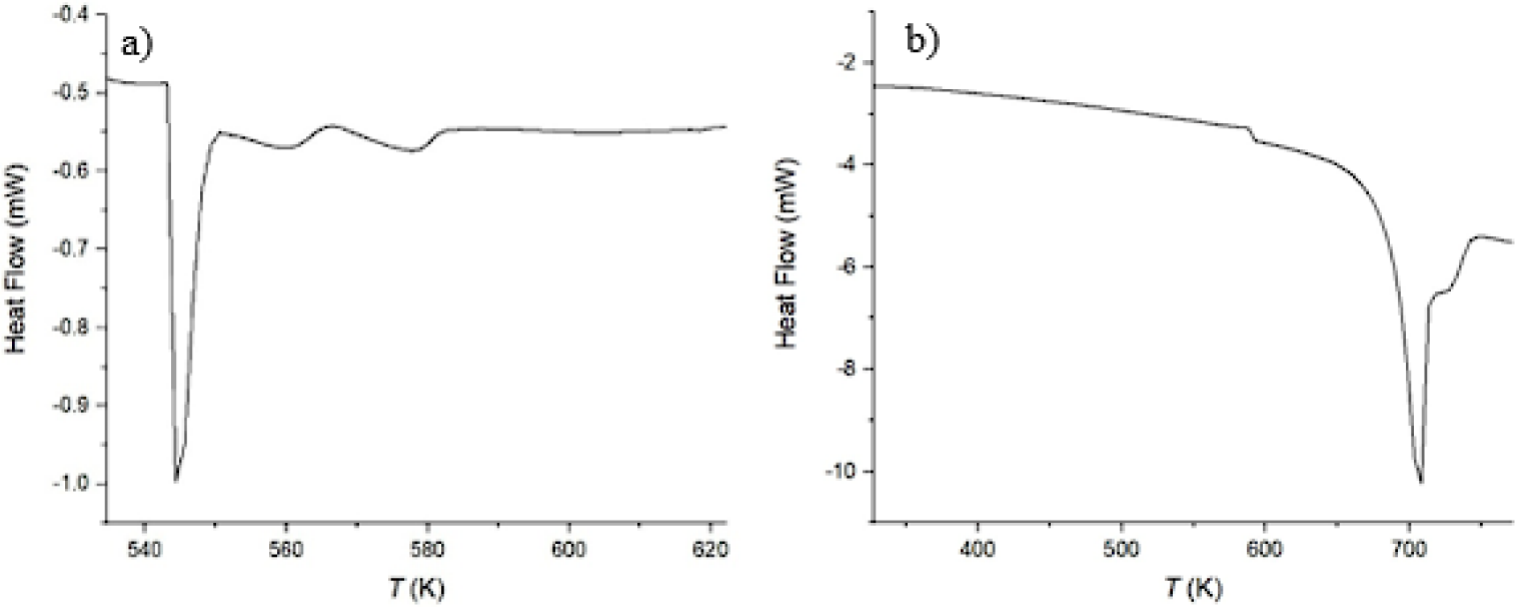
DSC thermogram sealed crucible a) 3,4-DIA b) 3,5-DIA.

Thermogravimetric analysis coupled with DSC was performed to evaluate the mass loss of the DIAs as a function of temperature (Figure 3). A small mass loss is observed before melting, likely due to sublimation. The DSC and TGA results further indicate that thermal decomposition begins upon melting and proceeds gradually thereafter.

**Figure 3:**
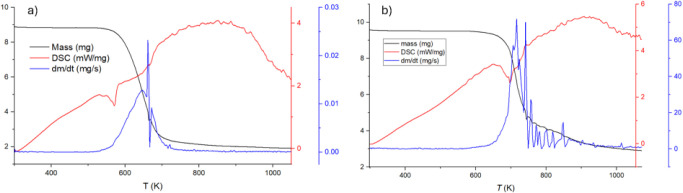
TGA thermogram a) 3,4-DIA b) 3,5-DIA.

The temperature dependence of the constant-pressure heat capacities of the reactants and synthesized compounds was calculated using the B3LYP/6-311++G­(d,p) method over the temperature range 296–470 K. The structures obtained by this function were considered the most stable identified conformers; the bond distances are reported in Figures S9 and S10, while the *C*_p_ values are found in Tables S1 to S5. Finally, the third-order polynomial fit (R^2^ = 0.9999) for the *C*_p_ is shown in eqs 3 to 7.

3 $$C_{p,m}\left(3,\mathrm{4‐DABA},g\right)/\left({\mathrm{J mol}}^{\mathrm{-1}}K^{\mathrm{-1}}\right)=-2.29\times 10^{-7}{\left(T/K\right)}^{3}-1.54\times 10^{-4}{\left(T/K\right)}^{2}+0.63\left(T/K\right)+0.59$$

4 $$C_{p,m}\left(3,\mathrm{5‐DABA},g\right)/\left({\mathrm{J mol}}^{\mathrm{-1}}K^{\mathrm{-1}}\right)=\mathrm{-1.}11\times 10^{\mathrm{-7}}{\left(T/K\right)}^{3}\mathrm{-2.}89\times 10^{\mathrm{-4}}{\left(T/K\right)}^{2}+0.68\left(T/K\right)+1.10$$

5 $$C_{p,m}\left(3,\mathrm{4 DIA},g\right)/\left({\mathrm{J mol}}^{\mathrm{-1}}K^{\mathrm{-1}}\right)=9.00^{\mathrm{-8}}{\left(T/K\right)}^{3}\mathrm{-1.}10\times 10^{\mathrm{-3}}{\left(T/K\right)}^{2}+1.84\left(T/K\right)-56.68$$

6 $$C_{p,m}\left(3,\mathrm{5DIA},g\right)/\left({\mathrm{J mol}}^{\mathrm{-1}}K^{\mathrm{-1}}\right)=9.00^{\mathrm{-8}}{\left(T/K\right)}^{3}\mathrm{-1.}10\times 10^{\mathrm{-3}}{\left(T/K\right)}^{2}+1.83\left(T/K\right)-55.38$$

7 $$C_{p,m}\left(\mathrm{PA},g\right)/\left({\mathrm{J mol}}^{\mathrm{-1}}K^{\mathrm{-1}}\right)=\mathrm{-4}.43^{\mathrm{-7}}{\left(T/K\right)}^{3}+1.51\times 10^{\mathrm{-4}}{\left(T/K\right)}^{2}+0.44\left(T/K\right)+1.72$$

### 4.2 Theoretical Results

DFT optimizations allowed the identification of the most stable conformers of the four compounds studied, enabling a comparison of their relative stability based on substituent positions (Figure 4). The detailed relative energies of all explored conformers are provided in the Supporting Information (Table S6), justifying the selection of the discussed global minima. It is worth noting that for 3,5-DIA, an extensive conformational search was not necessary, as its symmetry renders the structural orientations equivalent.

**Figure 4:**
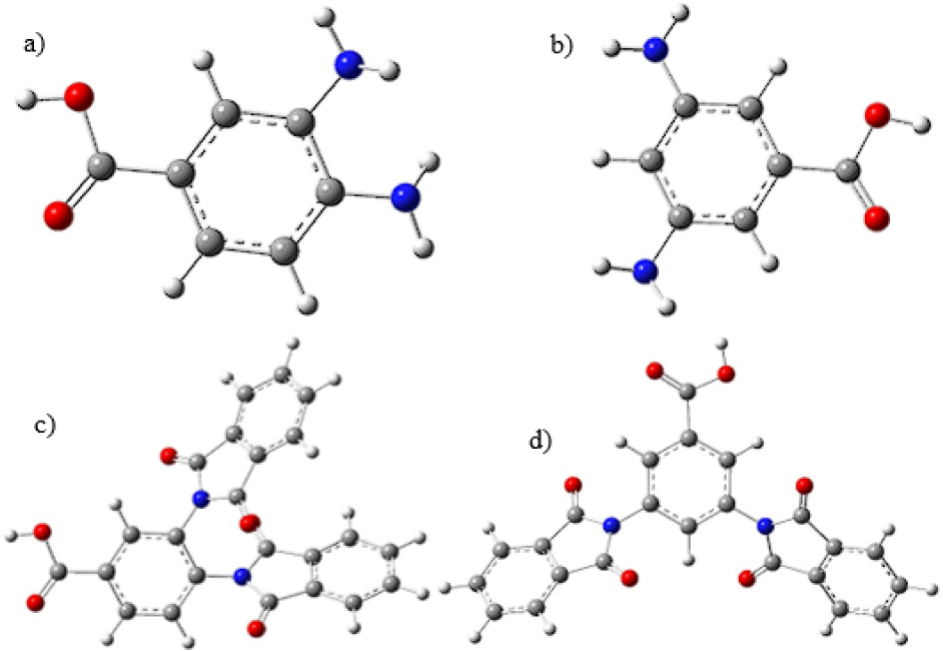
Front view of the B3LYP/6-311++G­(d,p) optimized structures of the a) 3,4-DABA, b) 3,5-DABA, c) 3,4-DIA and d) 3,5-DIA.

For the DABA isomers, thermochemical results show that 3,4-DABA is thermodynamically more stable than 3,5-DABA. Specifically, the sum of the zero-point corrected electronic energy (E+ZPE) at the B3LYP level for 3,4-DABA is −531.555319 au, while for 3,5-DABA it is −531.553658 au Consistently, the Gibbs free energies at 298.15 K are −531.590360 au and −531.588986 au, respectively, corresponding to a stabilization of the 3,4-DABA isomer by approximately 3.6 kJ·mol^–1^.

To move beyond geometric speculation and quantitatively determine the physical origins of this energy preference, a Natural Bond Orbital (NBO) analysis was conducted. The second-order perturbation theory analysis of the Fock matrix provides crucial insights into the stabilization mechanisms. Interestingly, the NBO results indicate that classical intramolecular hydrogen bonding between the adjacent amino groups in 3,4-DABA is negligible. Instead, the thermodynamic stability is driven by strong donor–acceptor conjugation interactions (Figure 5). Specifically, the lone pairs of the two nitrogen atoms strongly delocalize into the adjacent aromatic π* system (n→π* interactions). The stabilization energies (*E* ^2^) for these conjugations are 17.48 and 20.38 kcal/mol for each respective amino group, providing a substantial combined electronic stabilization of 37.86 kcal/mol. In contrast, the spatial separation of the functional groups in 3,5-DABA restricts this specific cooperative electronic delocalization, leading to slightly higher free energy.

**Figure 5:**
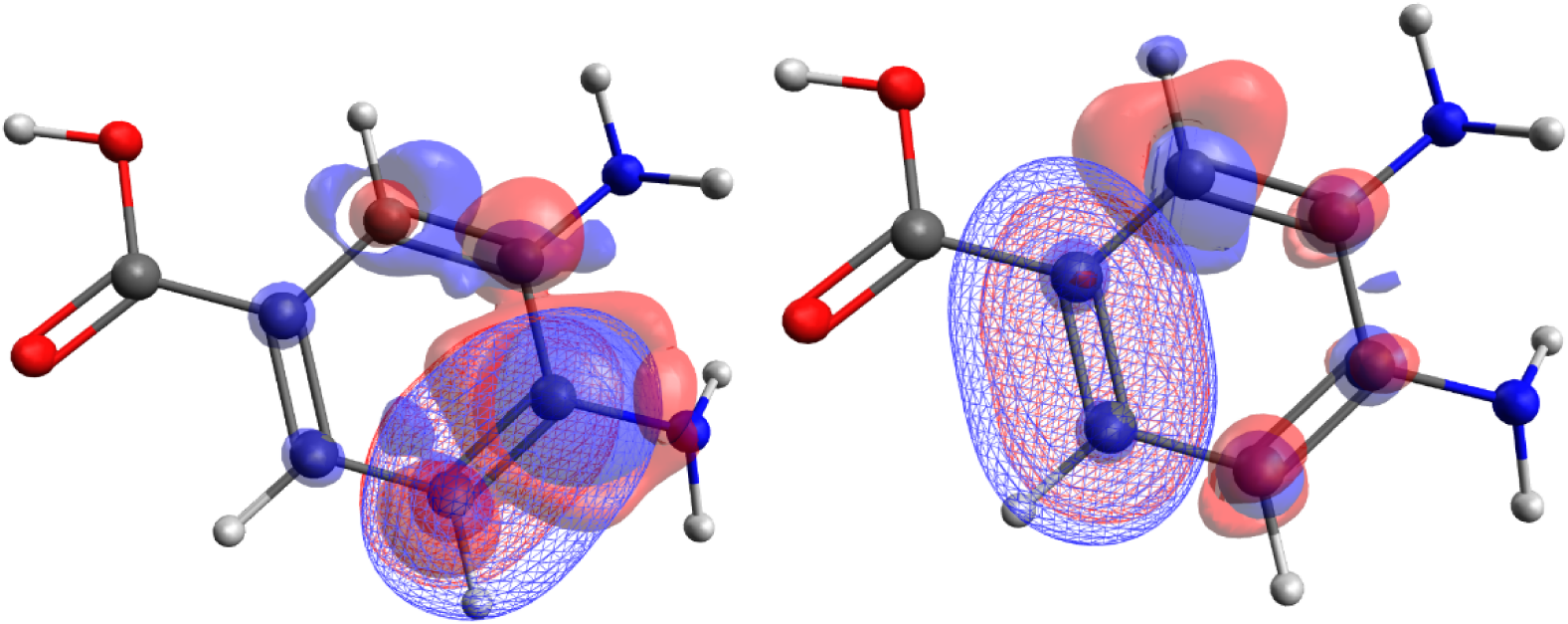
3D representation of the selected Natural Bond Orbitals (NBOs) for 3,4-DABA, illustrating the n→π* donor–acceptor overlap. Isosurface value = 0.04 au.

For the DIA compounds, a complementary trend is observed: the zero-point corrected electronic energy favors 3,5-DIA (E+ZPE = −1435.829815 au) over 3,4-DIA (E+ZPE = −1435.826868 au). The comparison of Gibbs free energies at 298.15 K confirms that 3,5-DIA (−1435.884461 au) is more stable than 3,4-DIA (−1435.880427 au) by approximately 10.6 kJ·mol^–1^.

Once the conformers with the lowest energy were found and analyzed, calculations were performed using composite methods to determine the enthalpy of formation for both the reactants and products to understand how the four compounds behave at a macroscopic level. Table 1 shows the energies obtained for G3, G4, and CBS-QB3 for the DABA isomers, while for DIAs, the values obtained by G3 (MP2) are shown. Table 2 shows the gas-phase enthalpy of formation values corrected to 298.15 K for all compounds.

**Table 1:** Calculated Energies at 0 K for the Compounds Studied by the G3, G4, CBS-QB3, and G3­(MP2) Levels of Theory^a^

	G3//G3(MP2)		G4		CBS-QB3	
Compound	*E*_0_	*H*_298_	*E*_0_	*H*_298_	*E*_0_	*H*_298_
3,4-DABA	–531.195461	–531.18434	–531.292586	–531.281442	–530.716844	–530.706013
3,5-DABA	–531.193292	–531.181919	–531.289927	–531.278535	–530.714067	–530.702933
3,4-DIA	–1442.54848	–1442.52315	-	-	-	-
3,5-DIA	–1442.54312	–1442.517686	-	-	-	-

a Hartree (E_h_) = 2625.46 kJ mol^–1^.

**Table 2:** Gas-Phase Enthalpies of Formation Using G3, G4, CBS-QB3, and G3­(MP2) Corrected to 298.15 K in kJ mol^–1^

Compound	G3	G4	CBS-QB3	G3(MP2)
3,4-DABA	–296.3	–298.8	–301.2	-
3,5-DABA	–289.9	–291.1	–293.1	-
3,4-DIA	-	-	-	–679.3
3,5-DIA	-	-	-	–665.0

As can be seen in Table 2, both the Gn and CBS methods show similar enthalpy values for the two DABA isomers, with a maximum difference of 4.9 kJ mol^–1^ corresponding to the G3 and CBS-QB3 methods. These values coincide with the maximum deviation of each model previously reported.^40^ The initial impression highlights the greater stability of the 3,4-DABA compound compared to its isomer. This, as mentioned earlier, is due to the delocalization of the nitrogen’s lone pairs of electrons. On the other hand, for the synthesized compounds, there appears to be no relationship between enthalpy and electronic energies, as 3,4-DIA seems to have greater stability. To contrast both results, Table 3 shows the absolute entropies, entropies of formation, and Gibbs free energies of formation for each compound. In the case of the DABA isomers, the enthalpy of formation value used was the average of the three calculations performed.

**Table 3:** Thermochemical Properties of Molecules

Compound	Δ_f_ *H*° (g)/kJ mol^–1^	*S*° (g)/ J mol^–1^ K^–1^	Δ_f_ *S*°(g)/J mol^–1^ K^–1^	Δ_f_ *G*° (g)/kJ mol^–1^
H_2_O	-241.83 ± 0.04^a^	188.84 ± 0.01^a^	-44.25 ± 0.01	-228.64 ± 0.04
PA	–371.69^b^	365.1	–249.5	–297.3
3,4-DABA	–298.8	403.8	–555.2	–133.3
3,5-DABA	–291.4	408.8	–550.2	–127.4
3,4-DIA	–679.3	675.2	–1046.9	–367.2
3,5-DIA	–665.0	685.7	–1036.3	–356.0

a Taken from ref 41.

b Taken from ref 42.

For the highly functionalized DIA compounds, a rigorous macroscopic thermochemical evaluation at the G3­(MP2) level confirms that the 3,4-DIA isomer is thermodynamically preferred. The calculated standard enthalpy of formation (Δ_f_ *H*°) is considerably more exothermic for 3,4-DIA (−679.3 kJ mol^–1^) compared to 3,5-DIA (−665.0 kJ mol^–1^). Utilizing the absolute entropies consistently derived from the G3­(MP2) methodology, the standard Gibbs free energies of formation (Δ_f_ *G*°) at 298.15 K are −367.2 kJ mol^–1^ for 3,4-DIA and −356.0 kJ mol^–1^ for 3,5-DIA. This demonstrates a robust thermodynamic stabilization of 11.2 kJ mol^–1^in favor of the 3,4-DIA configuration.

The physical justification for this stability highlights a delicate balance between localized electronic delocalization and global structural compactness. The NBO analysis reveals that the dominant stabilizing electronic interactions arise from the conjugation of the imide nitrogen lone pairs into the adjacent carbonyl π* orbitals (n→π*_C=O_). In 3,5-DIA, the spatial separation of the imide rings eliminates steric clashes, allowing for optimal coplanarity and slightly superior localized conjugation (*E* ^2^ totaling 96.15 kcal mol^–1^ for N10). In 3,4-DIA (Figure 6), the proximity of the imide groups induces minor steric hindrance, leading to a slightly asymmetrical and reduced local conjugation (*E* ^2^ totaling 90.39 kcal mol^–1^ for N8). However, this minor loss in localized n→π* stabilization is overwhelmingly compensated by a massive enthalpic advantage. The 3,4-positioning enforces a compact molecular packing that promotes strong long-range stabilizing forces, such as favorable dipole–dipole alignments and dispersive interactions between the rigid diimide fragments. Consequently, macroscopic compactness dictates the final thermodynamic stability, favoring the 3,4-DIA isomer.

**Figure 6:**
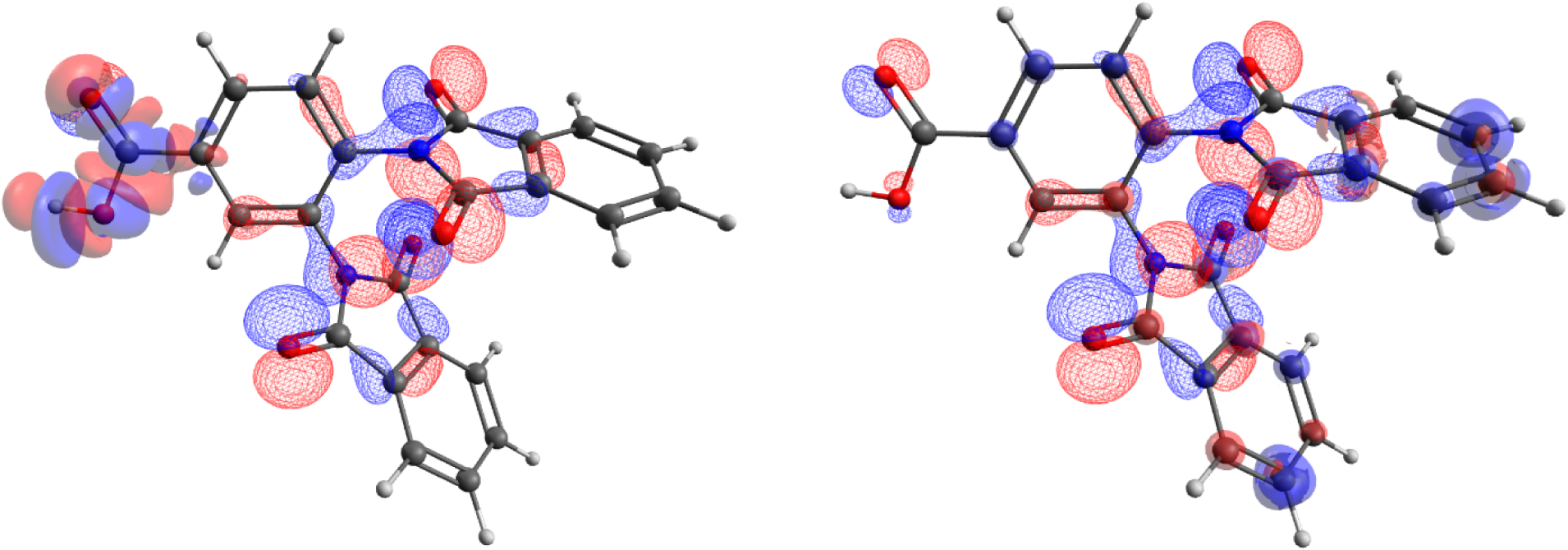
3D representation of the selected Natural Bond Orbitals (NBOs) for 3,4-DIA, illustrating the n→π*_C=O_ donor–acceptor overlap. Isosurface value = 0.04 au.

To characterize these stabilizing forces, a topological analysis of the electron density was conducted using the Quantum Theory of Atoms in Molecules (QTAIM) via the Multiwfn 3.8 software. The molecular graphs reveal distinct differences in the electronic connectivity of the isomers. In 3,4-DIA (Figure 7), two primary (3,-1) bond critical points (BCPs) were identified between nitrogen and oxygen atoms: N8–O31 (ρ = 0.0129 au, ∇ ^2^ρ = 0.0470 au) and N21–O18 (ρ = 0.0130 au, ∇ ^2^ρ = 0.0473 au). The negative values of the potential energy density *V*(r) at these BCPs (−0.0091 and −0.0092 au, respectively) indicate that these interactions are fundamentally attractive and play a crucial role as structural “anchors” that enforce a compact, energetically preferred conformation.

**Figure 7:**
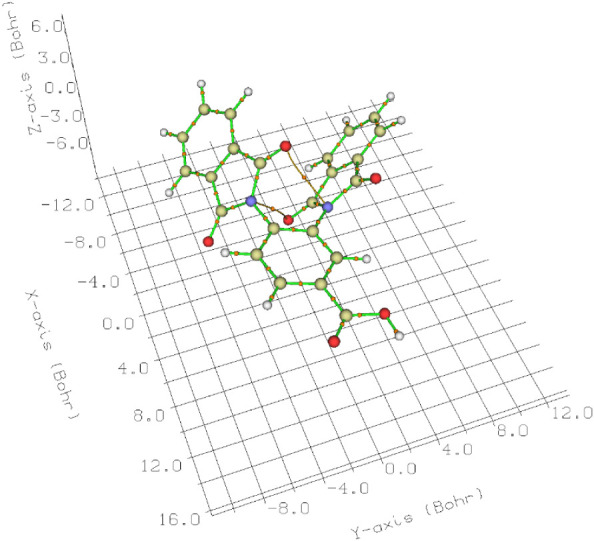
Molecular graph of 3,4-DIA showing the BCP (3,-1) between N and O.

In contrast, the topological map of 3,5-DIA (Figure 8) reveals a more fragmented distribution of critical points. We identified multiple BCPs involving oxygen–hydrogen and oxygen–carbon interactions, such as O31–H34 (ρ = 0.0114 au) and O23–C5 (ρ = 0.0110 au). While these BCPs also exhibit negative potential energy density, their lower electron density and the spatial dispersion of these points suggest that 3,5-DIA lacks the robust, localized electronic stabilization provided by the N–O interactions in the 3,4-isomer.

**Figure 8:**
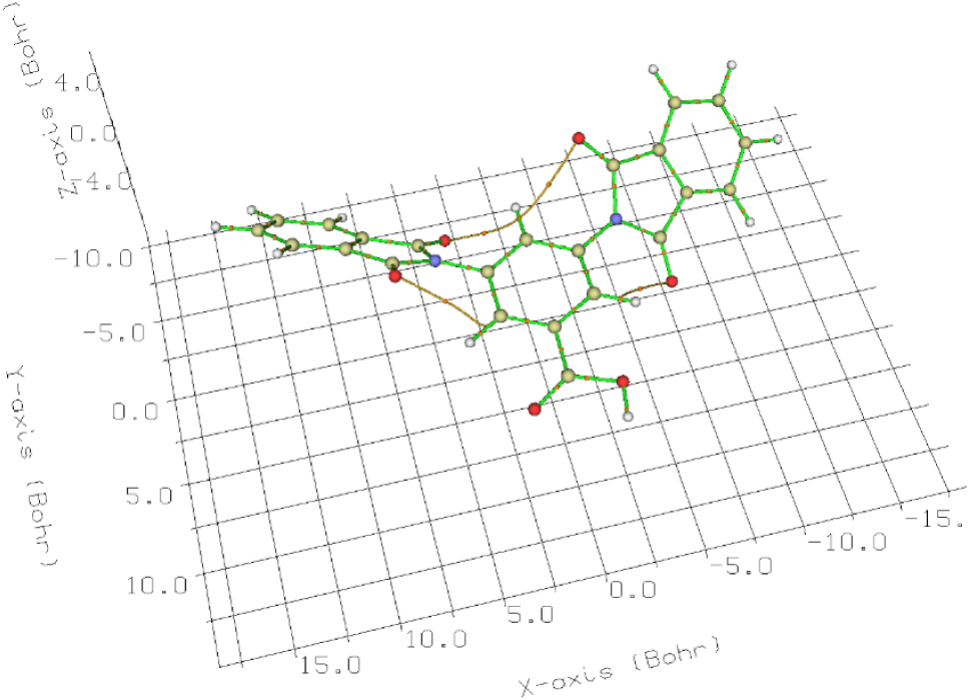
Molecular graph of 3,5-DIA showing the absence of the N–O stabilizing path and presence of dispersed interactions.

Consequently, the thermodynamic stability of 3,4-DIA is not merely a product of electronic conjugation but a synergistic outcome of macroscopic structural compactness. The QTAIM results confirm that the 3,4-substitution pattern promotes a “folded” architecture stabilized by significant intramolecular attractions, whereas the 3,5-analogue exhibits an “open” geometry characterized by weaker, noncooperative interactions.

As shown in Figure 9, the 3,4-substituted isomers are thermodynamically more stable than their corresponding 3,5-substituted counterparts. This trend is reflected in the gas-phase standard enthalpies of formation, where the isomerization enthalpies amount to 7.4 and 14.3 kJ·mol^–1^ for the DABA and DIA pairs, respectively. The same stability ordering was predicted by the topological analysis and ωB97XD/6-311++G­(d,p) electronic energies, which indicate that 3,4-DIA (−1444.503785 au) is more stable than 3,5-DIA (−1444.502242 au) by 4.05 kJ·mol^–1^.

**Figure 9:**
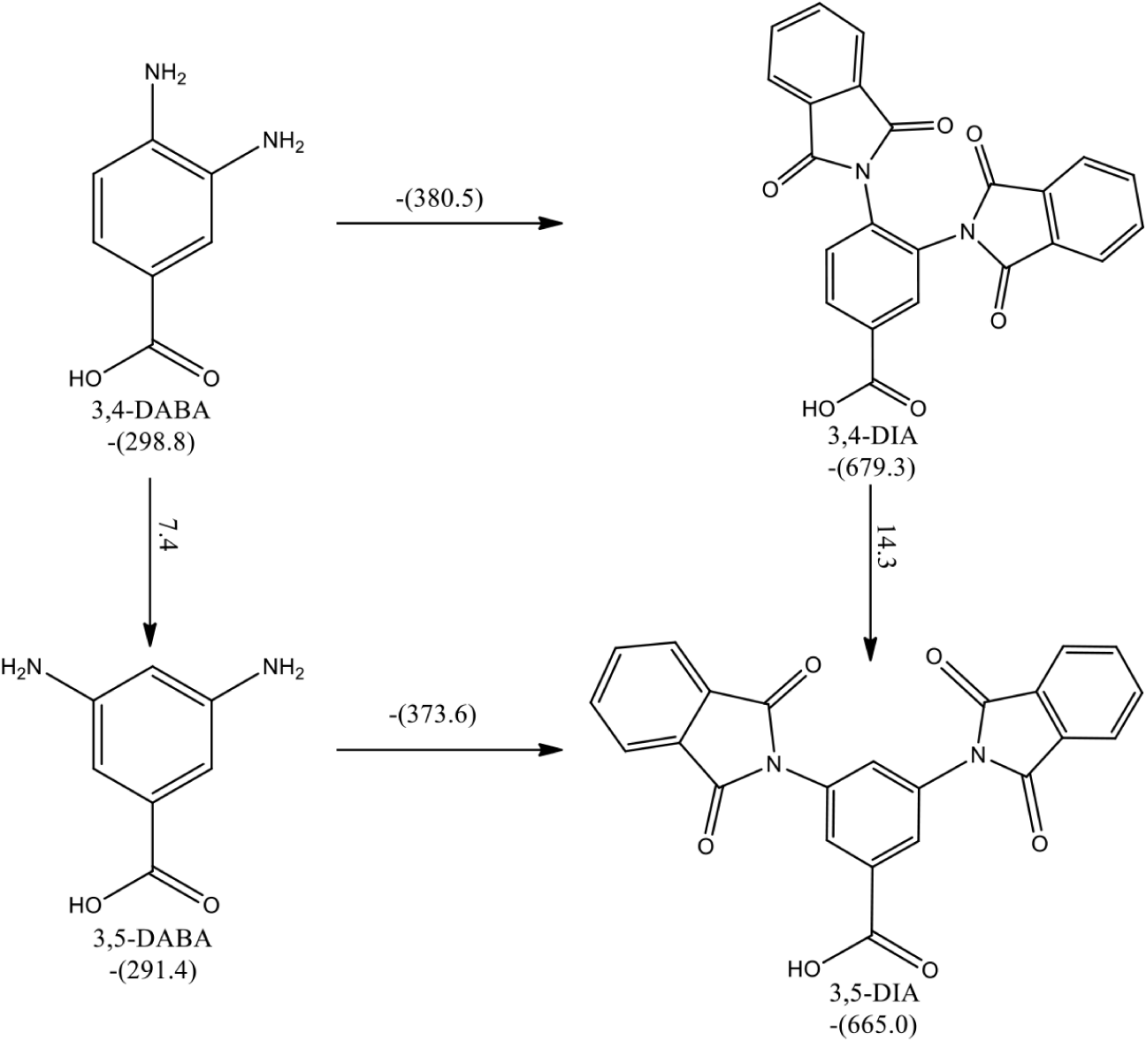
Enthalpic increases in kJ mol^–1^ for each isomeric pair.

Figure 9 also shows that the enthalpy changes associated with the conversion of DABA into DIA are very similar for both substitution patterns, yielding values of −380.5 and −373.6 kJ·mol^–1^ for the 3,4- and 3,5-isomers, respectively. The difference between these values (6.9 kJ·mol^–1^) is close to the isomerization enthalpy calculated for the DABA pair (7.4 kJ·mol^–1^), indicating that the energetic preference for the 3,4-substitution pattern already exists in the DABA precursors and is retained after their conversion into the corresponding DIA derivatives. Furthermore, the larger isomerization enthalpy observed for the DIA pair (14.3 kJ·mol^–1^) suggests that the formation of the DIA framework further enhances the thermodynamic preference for the 3,4-substituted isomer. Therefore, both the topological descriptors and the thermochemical results consistently point to a greater intrinsic stabilization of the 3,4-isomers relative to their corresponding 3,5-isomers.

#### 4.2.1 Equilibrium Reaction for the Synthesis of the DIAs in the Gas Phase

Using the values of Δ_f_ *H*°_m_(g) and Δ_f_ *S*°_m_(g) reported in Table 3, the equilibrium constant (*K°*), standard reaction enthalpy (Δ_r_ *H*°), standard reaction entropy (Δ_r_ *S*°), and standard Gibbs free energy (Δ_r_ *G*°) for the formation of 3,4- and 3,5-DIA (Figure 1) in the gas phase at 298.15 K were determined. The calculated thermodynamic parameters are presented in Table 4.

**Table 4:** Values Calculated for the Reaction of DIAs at 298.15 K

Compound	Δ_r_ *H*°_m_(g), kJ mol^–1^	Δ_r_ *S*°_m_(g), J mol^–1^ K^–1^	Δ_r_ *G*°_m_(g), kJ mol^–1^	*K°*
3,4-DIA	–120.76	–81.12	–96.57	8.32 × 10^16^
3,5-DIA	–113.86	–75.62	–91.31	9.96 × 10^15^

Both reactions are clearly exothermic, as evidenced by the negative Δ_r_ *H*°_m_(g) values. The formation of 3,4-DIA is slightly more exothermic than that of 3,5-DIA, suggesting a greater enthalpic stabilization for the former isomer. Although both reactions exhibit negative Δ_r_ *S*°_m_(g) values, indicating a decrease disordered upon formation, the strongly negative Δ_r_ *G*°_m_(g) values confirm that the processes are thermodynamically favorable at 298.15 K.

The magnitude of the equilibrium constants (10^15^–10^16^) demonstrates that, under gas-phase standard conditions, the equilibrium is overwhelmingly shifted toward the formation of both DIAs, with a modest thermodynamic preference for 3,4-DIA.

## 5 Conclusions

In this study, a solvent-free solid-state route for the synthesis of the 3,4- and 3,5-DIA isomers was successfully developed, demonstrating a simple and environmentally friendly approach consistent with green chemistry principles. The methodology provides an efficient pathway for the synthesis of bisphthalimide derivatives while reducing solvent consumption and simplifying experimental procedures.

Structural characterization by FTIR, ^1^H NMR, and mass spectrometry confirmed the formation of the target compounds. Thermal analysis using DSC and TGA revealed differences in thermal stability and phase transitions between the isomers, indicating that the substitution pattern plays a significant role in their thermal behavior.

Quantum chemical calculations were employed to predict thermochemical properties and complement the experimental results. To the best of our knowledge, the thermochemical properties reported for the 3,4-/3,5-DABA and 3,4-/3,5-DIA isomeric pairs have not been previously described in the literature.

The calculated thermochemical data provided valuable information for evaluating the reaction equilibrium properties and contributed to a more in-depth understanding of the energetic relationships between reactants and products. The combined experimental and theoretical approach provided new insight into the relationship between molecular structure and thermochemical behavior. Overall, the results demonstrate that solvent-free solid-state synthesis is an effective strategy for the preparation and study of structurally related bisphthalimide isomers.

## Supplementary Material



## Notes

Infrared (FTIR) spectra; ^1^H NMR spectra; mass spectra (MS) and proposed fragmentation paths; B3LYP/6-311++G­(d,p) optimized geometries; temperature-dependent molar heat capacities (*C*_p,m_ (g)); relative electronic energies for the evaluated conformers (PDF)
