# Supplementary material for: Green Synthesis, Characterization, and the Prediction of Its Thermochemical Properties of the Isomers 3,4- and 3,5-Bis(1,3-dihydro-1,3-dioxo-2H-isoindol-2-yl)benzoic Acid
Source: ACS Omega. 2026 Jul 30;11(31):46777–86. doi: 10.1021/acsomega.6c04687 (PMC13470730; doi:10.1021/acsomega.6c04687)
Supplement: Supplementary file 1 [file ao6c04687_si_001.pdf]

GREEN SYNTHESIS, CHARACTERIZATION AND THE PREDICTION ITS THERMOCHEMICAL PROPERTIES OF THE ISOMERS 3,4 AND 3,5-BIS(1,3-DIHYDRO-1,3-DIOXO-2H-ISOINDOL-2-YL) BENZOIC ACID

Diego Espinosa-Morales[a], Fausto Díaz-Sánchez[a], María J. Sáenz de Miera-Ramos[a], Miguel A. García-Castro[a*], Luis H. Gutiérrez-Gámez[a], Francisco Paraguay-Delgado[b]

[a] Facultad de Ingeniería Química de la Benemérita Universidad Autónoma de Puebla, 18 Sur y Av. San Claudio, Puebla, Puebla C.P. 72570, México

[b] Centro de Investigación en Materiales Avanzados SC. Miguel de Cervantes 120, Complejo Industrial Chihuahua, Chihuahua, Chihuahua C.P. 31136, México

[*] Corresponding Author: miguel.garciacastro@correo.buap.mx

Supporting Information

List of contents

Figure S1. IR spectrum of 3,4-DIA

Figure S2. ^{1}H NMR spectrum (DMSO-d_6) of 3,4-DIA

Figure S3. Mass spectrum of 3,4-DIA

Figure S4. Fragmentation path of 3,4-DIA

Figure S5. IR spectrum of 3,5-DIA

Figure S6. ^{1}H NMR spectrum (DMSO-d_6) of 3,5-DIA

Figure S7. Mass spectrum of 3,5-DIA

Figure S8. Fragmentation path of 3,5-DIA

Figure S9. Optimized structure for 3,4-DIA obtained by B3LYP/6-311++G(d,p)

Figure S10. Optimized structure for 3,5-DIA obtained by B3LYP/6-311++G(d,p)

Table S1. Values calculated for *C*p of 3,4-DABA at different temperatures

Table S2. Values calculated for *C*p of 3,5-DABA at different temperatures

Table S3. Values calculated for *C*p of 3,4-DIA at different temperatures

Table S4. Values calculated for *C*p of 3,5-DIA at different temperatures

Table S5. Values calculated for *C*p of PA at different temperatures

Table S6. Electronic energies for the different conformers of the 3,4 and 3,5 DABA and DIA in Eh.

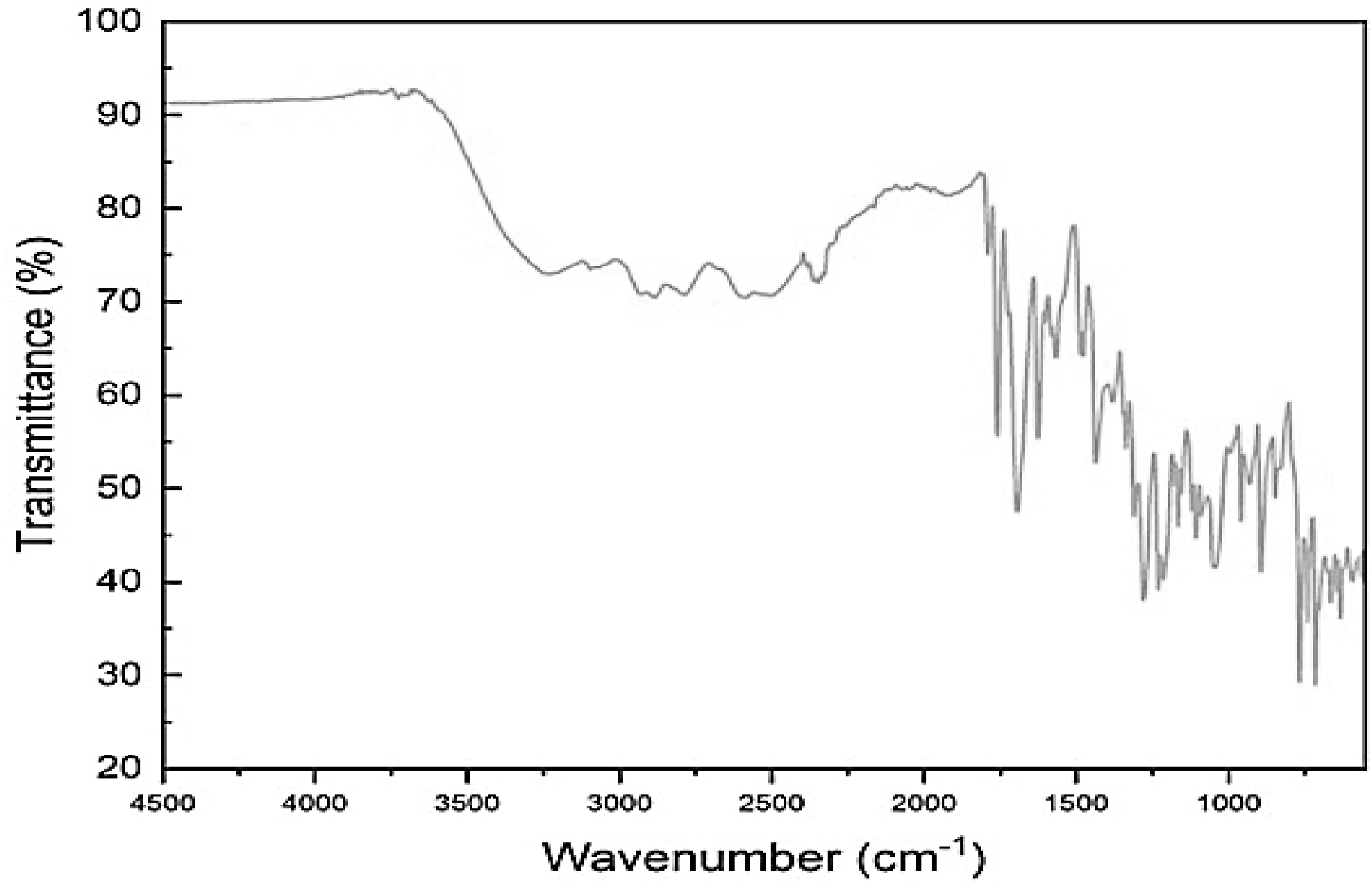

Figure S1. IR spectrum of 3,4-DIA

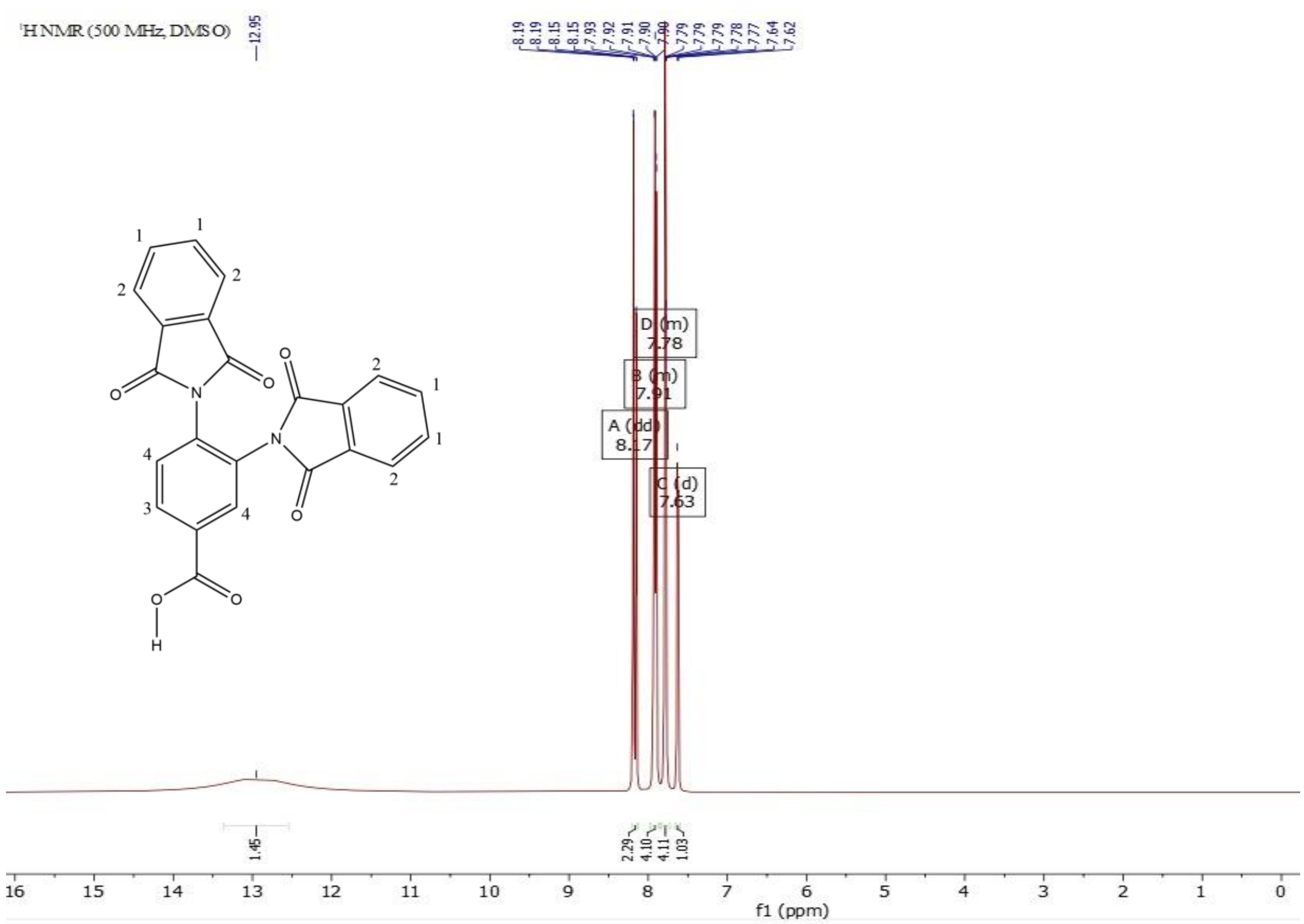

Figure S2. ^{1}H NMR spectrum (DMSO-d_6) of 3,4-DIA

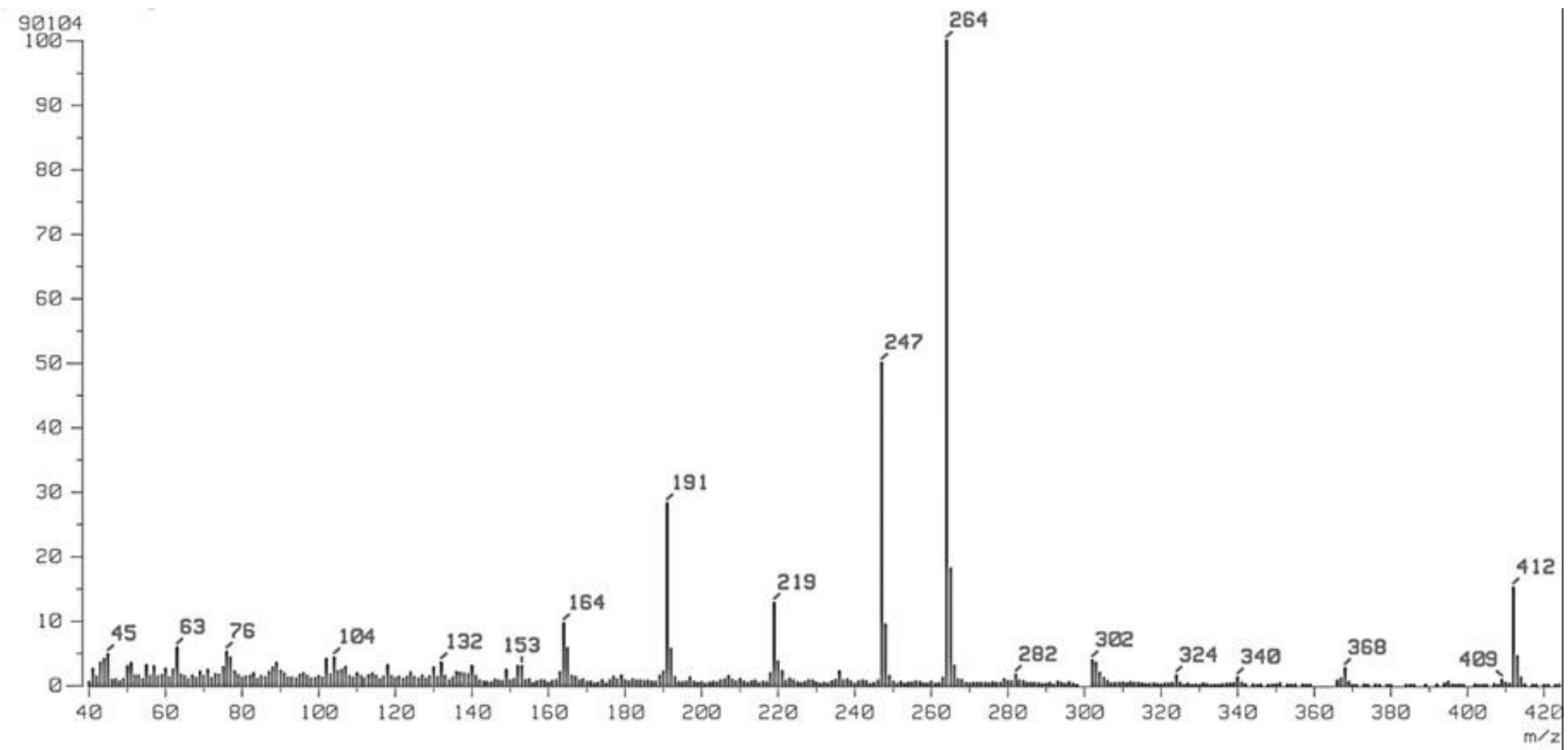

Figure S3. Mass spectrum of 3,4-DIA

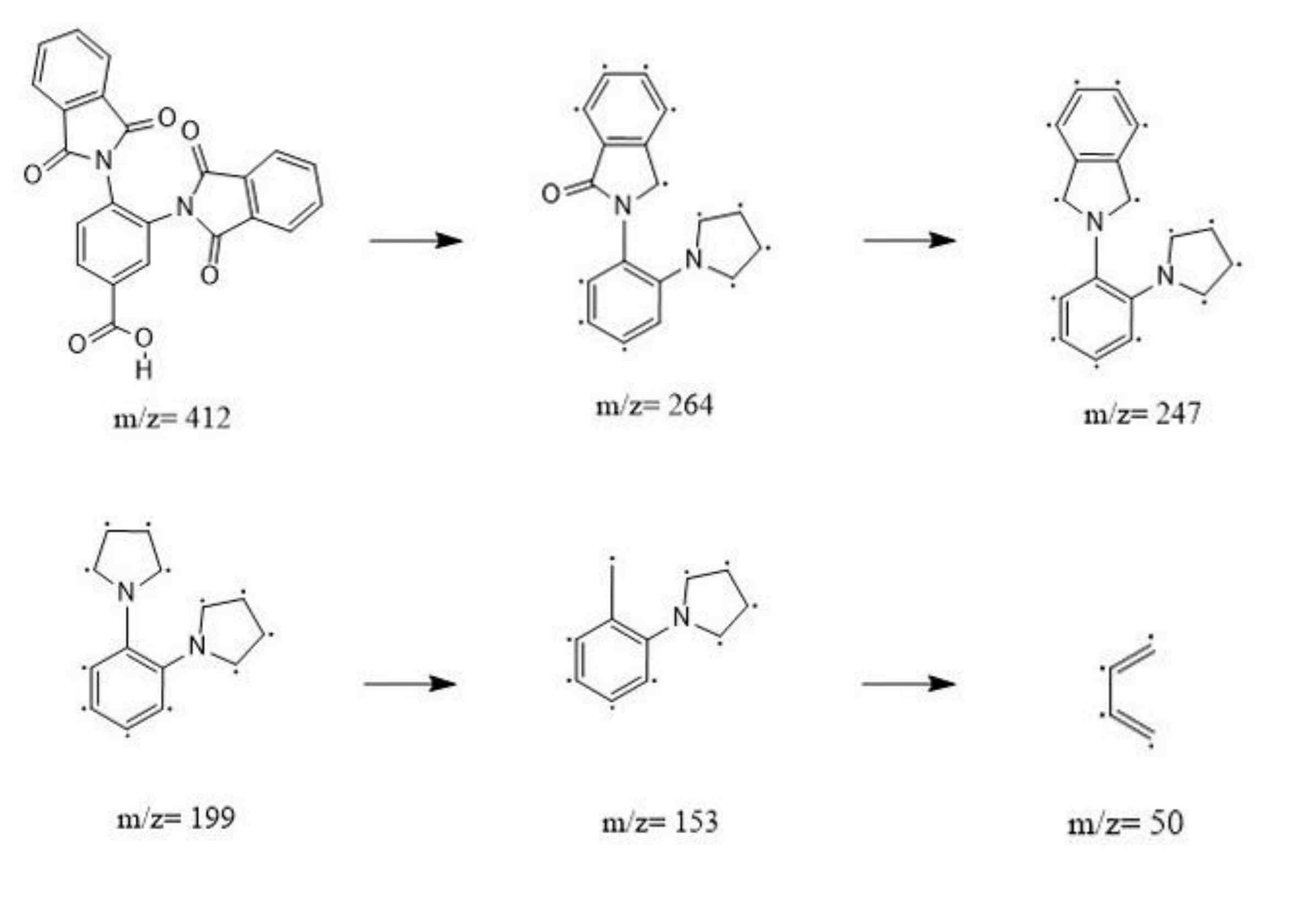

Figure S4. Fragmentation path of 3,4-DIA

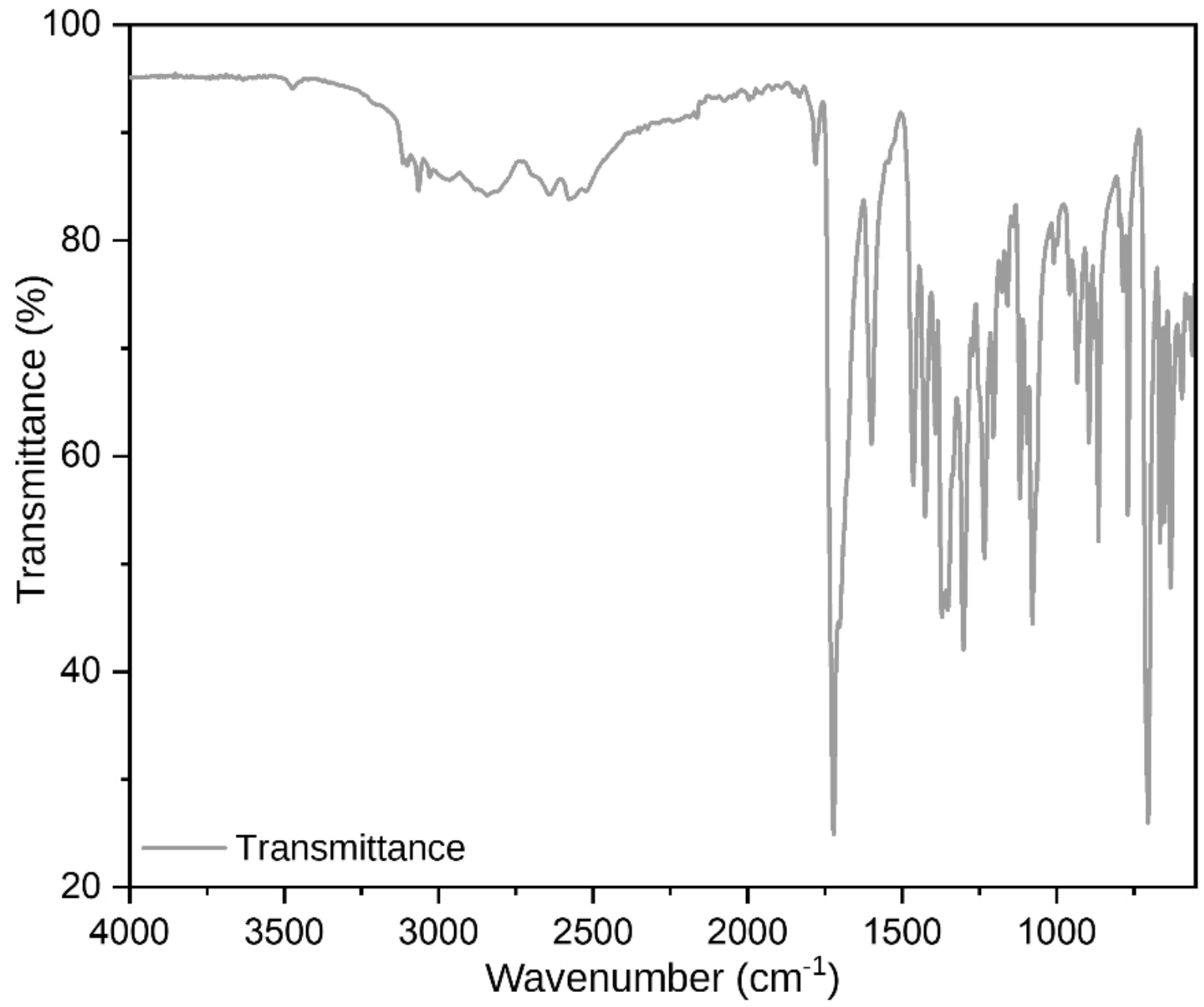

Figure S5. IR spectrum of 3,5-DIA.

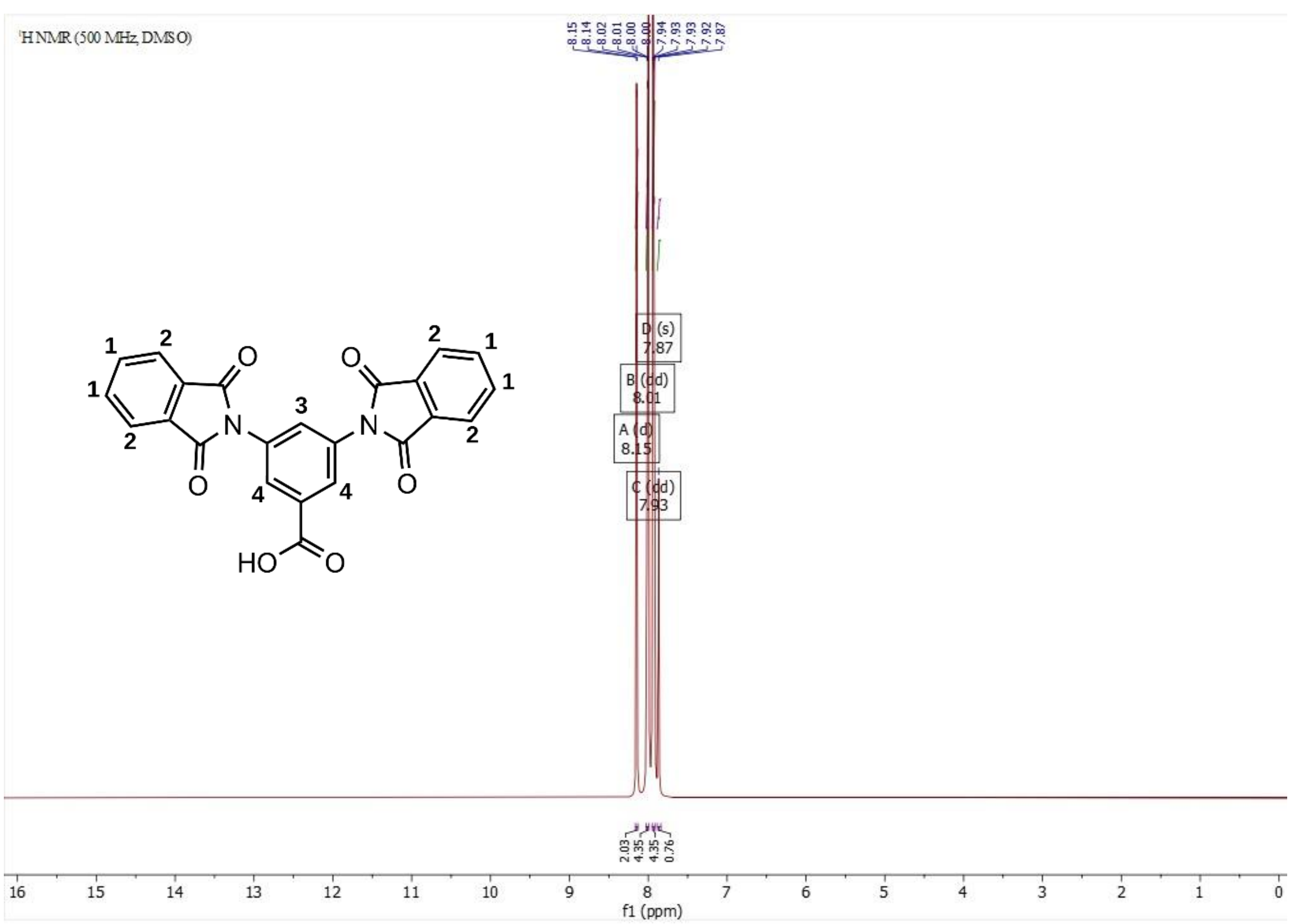

Figure S6. ^{1}H NMR spectrum (DMSO-d6) of 3,5-DIA.

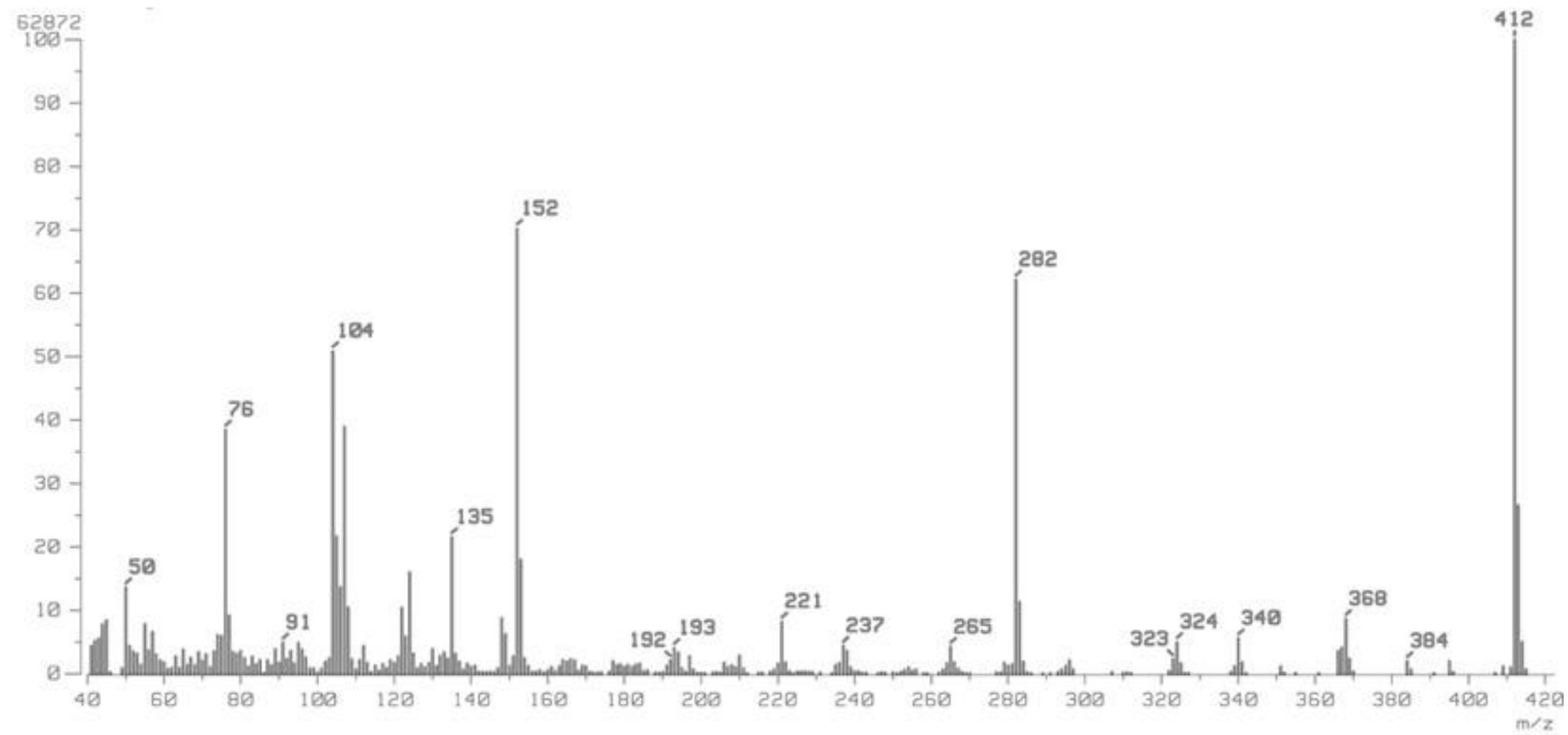

Figure S7. Mass spectrum of 3,5-DIA.

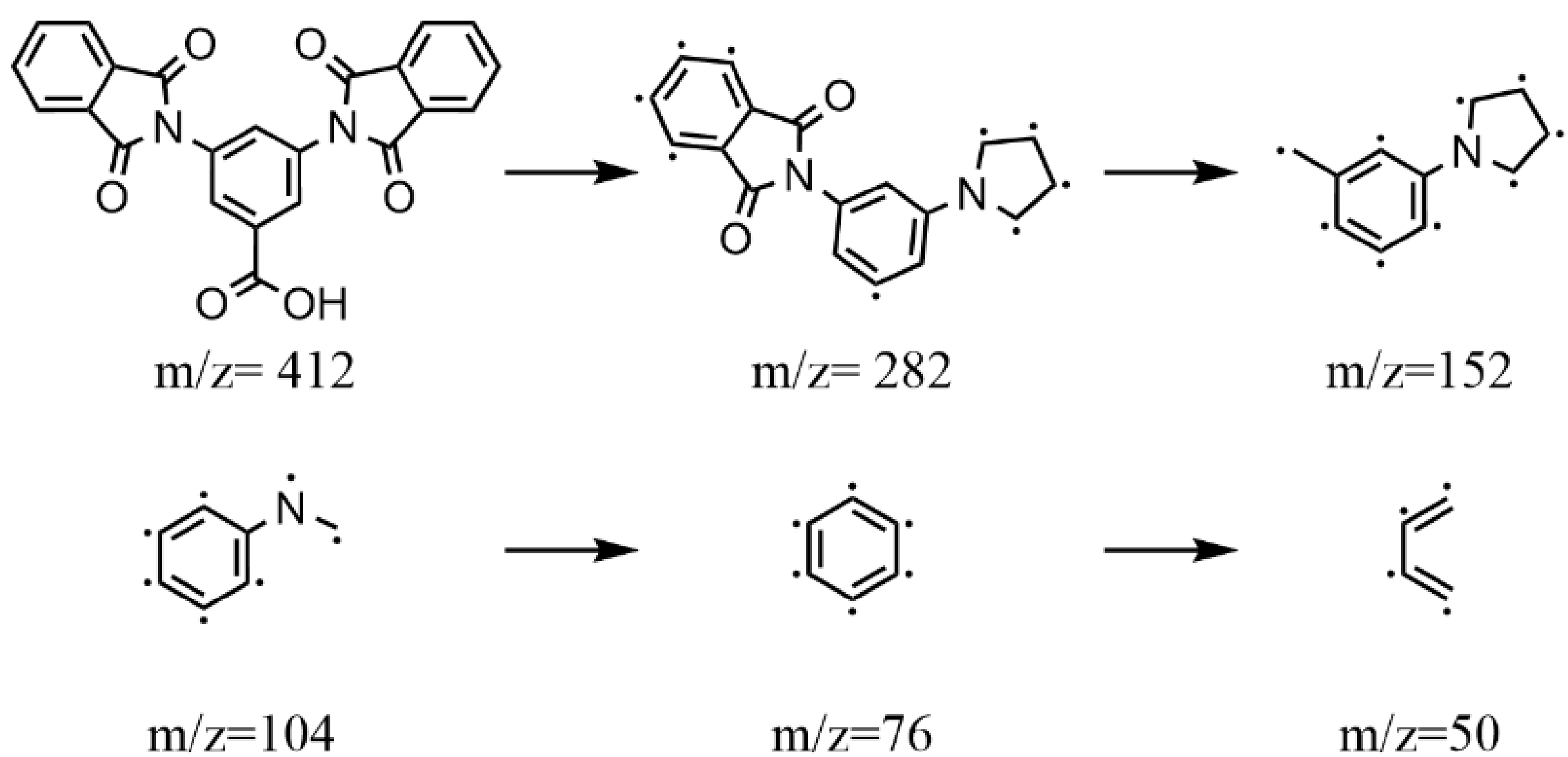

Figure S8. Fragmentation path of 3,5-DIA.

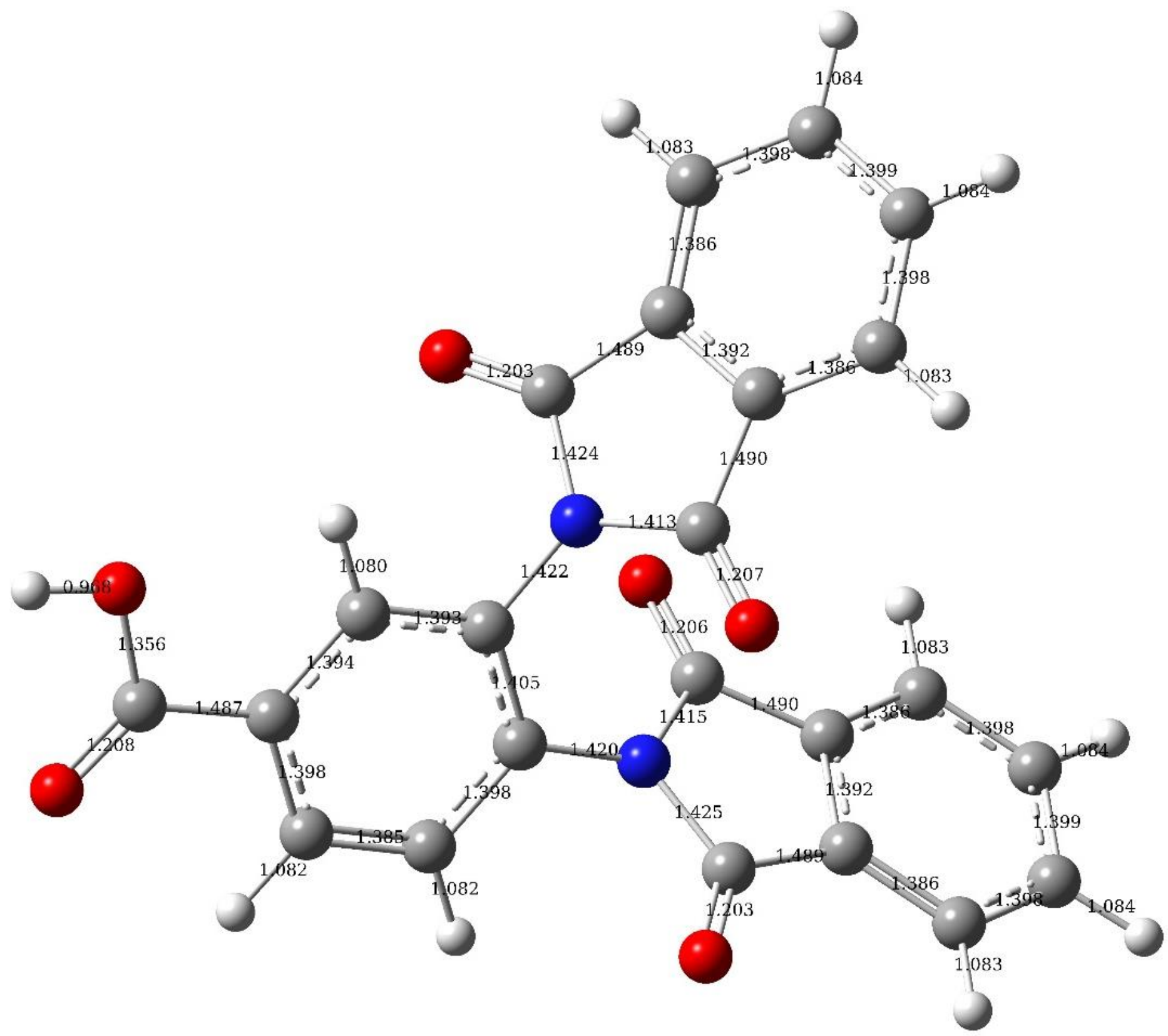

Figure S9. Optimized structure for 3,4-DIA obtained by B3LYP/6-311++G(d,p)

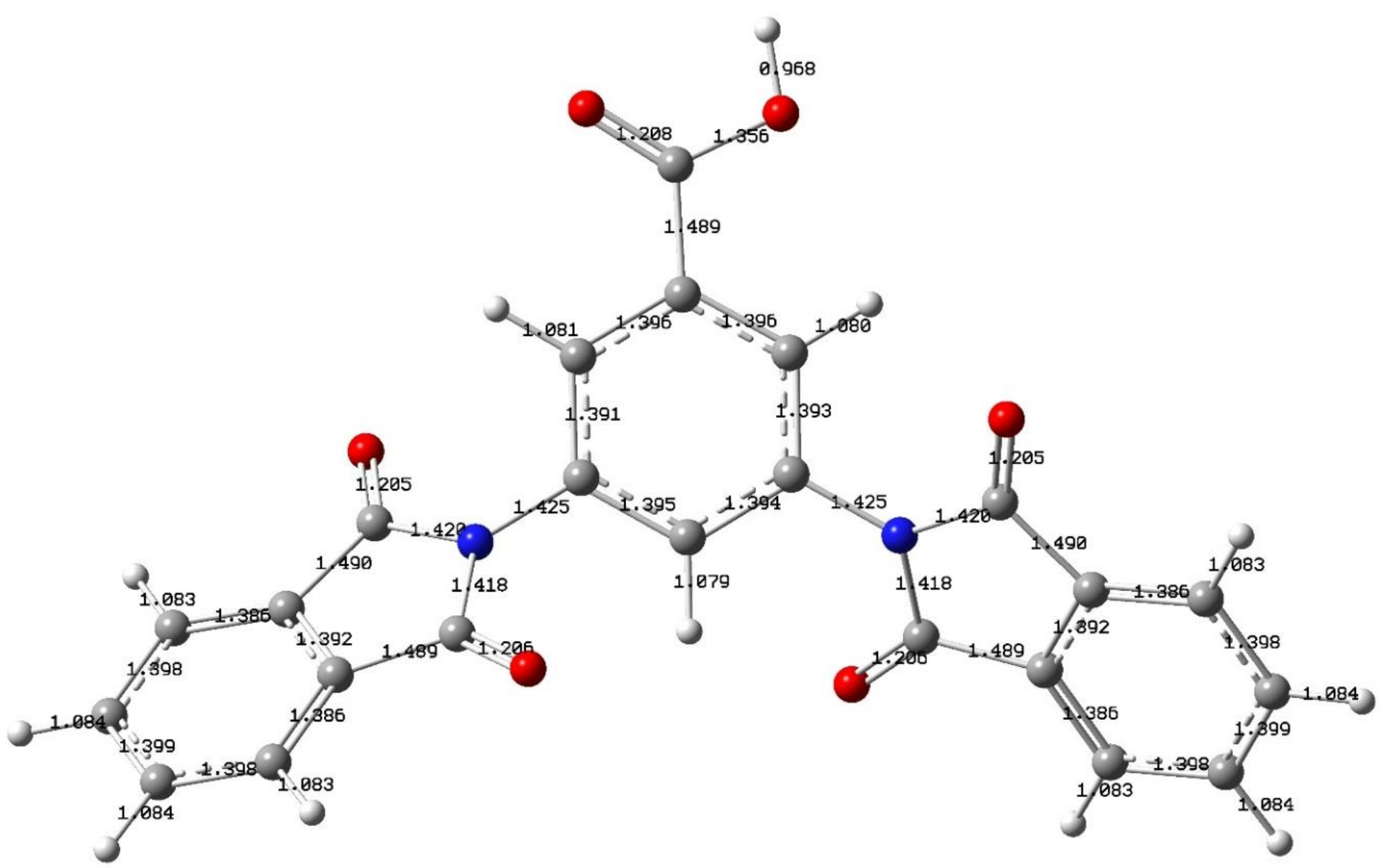

Figure. S10. Optimized structure for 3,5-DIA obtained by B3LYP/6-311++G(d,p).

Table S1

Values calculated for *C*p of 3,4-DABA at different temperatures

	$C_{p,m}$ (g)		$C_{p,m}$ (g)		$C_{p,m}$ (g)
T/K	J mol^{-1} K^{-1}	*T*/K	J mol^{-1} K^{-1}	*T*/K	J mol^{-1} K^{-1}
296	168.890328	340	189.420516	384	208.449562
298.15	169.92585	342	190.319099	386	209.27702
300	170.814349	344	191.214549	388	210.101175
302	171.772232	346	192.106852	390	210.922024
304	172.727333	348	192.995997	392	211.739564
306	173.679631	350	193.881972	394	212.553793
308	174.629102	352	194.764766	396	213.364711
310	175.575727	354	195.644369	398	214.172315
312	176.519484	356	196.52077	400	214.976606
314	177.460353	358	197.393959	402	215.777583
316	178.398314	360	198.263927	404	216.575246
318	179.333348	362	199.130665		
320	180.265437	364	199.994166		
322	181.194561	366	200.85442		
324	182.120704	368	201.711421		
326	183.043847	370	202.565162		
328	183.963974	372	203.415636		
330	184.881069	374	204.262837		
332	185.795115	376	205.106759		
334	186.706098	378	205.947397		
336	187.614002	380	206.784746		
338	188.518813	382	207.618802		

Table S2

Values calculated for *C*p of 3,5-DABA at different temperatures

	$C_{p,m}$ (g)		$C_{p,m}$ (g)		$C_{p,m}$ (g)
T/K	J mol^{-1} K^{-1}	*T*/K	J mol^{-1} K^{-1}	*T*/K	J mol^{-1} K^{-1}
296	173.282097	340	193.49843	384	212.158073
298.15	174.304797	342	194.380909	386	212.968369
300	175.181994	344	195.260154	388	213.775359
302	176.12739	346	196.136157	390	214.579043
304	177.069736	348	197.008914	392	215.379423
306	178.00902	350	197.878418	394	216.176498
308	178.945232	352	198.744663	396	216.97027
310	179.87836	354	199.607645	398	217.760741
312	180.808393	356	200.467358	400	218.547913
314	181.735321	358	201.323797	402	219.331787
316	182.659133	360	202.176959	404	220.112367
318	183.57982	362	203.026839		

320	184.497372	364	203.873433
322	185.411778	366	204.716739
324	186.323029	368	205.556752
326	187.231117	370	206.39347
328	188.136032	372	207.226892
330	189.037766	374	208.057013
332	189.93631	376	208.883833
334	190.831656	378	209.70735
336	191.723796	380	210.527563
338	192.612724	382	211.344471

Table S3

Values calculated for *C*p of 3,4-DIA at different temperatures

	$C_{p,m}$ (g)		$C_{p,m}$ (g)		$C_{p,m}$ (g)		$C_{p,m}$ (g)
T/K	J mol^{-1} K^{-1}	*T*/K	J mol^{-1} K^{-1}	*T*/K	J mol^{-1} K^{-1}	*T*/K	J mol^{-1} K^{-1}
296	394.065033	340	445.143658	384	493.025903	428	537.15622
298.15	396.621394	342	447.395383	386	495.115718	430	539.069119
300	398.816757	344	449.640344	388	497.197683	432	540.973879
302	401.185536	346	451.878471	390	499.271771	434	542.870504
304	403.549434	348	454.109694	392	501.337954	436	544.758995
306	405.908334	350	456.333949	394	503.396208	438	546.639355
308	408.262122	352	458.551171	396	505.446509	440	548.511591
310	410.610687	354	460.761298	398	507.488836	442	550.375706
312	412.95392	356	462.964271	400	509.523167	444	552.231707
314	415.291712	358	465.160031	402	511.549483	446	554.079603
316	417.623961	360	467.348523	404	513.567768	448	555.9194
318	419.950564	362	469.529694	406	515.578005	450	557.751109
320	422.27142	364	471.703491	408	517.58018	452	559.57474
322	424.586433	366	473.869866	410	519.574278	454	561.390302
324	426.895508	368	476.028768	412	521.560289	456	563.197809
326	429.198551	370	478.180154	414	523.538202	458	564.997273
328	431.495472	372	480.323979	416	525.508007	460	566.788708
330	433.786183	374	482.4602	418	527.469697	462	568.572126
332	436.070598	376	484.588778	420	529.423264	464	570.347544
334	438.348633	378	486.709673	422	531.368704	466	572.114977
336	440.620207	380	488.822848	424	533.306012	468	573.874442
338	442.885241	382	490.92827	426	535.235185	470	575.625955

Table S4

Values calculated for *C*p of 3,5-DIA at different temperatures

	$C_{p,m}$ (g)		$C_{p,m}$ (g)		$C_{p,m}$ (g)		$C_{p,m}$ (g)

T/K	J mol^{-1} K^{-1}	T/K	J mol^{-1} K^{-1}	T/K	J mol^{-1} K^{-1}	T/K	J mol^{-1} K^{-1}
296	394.599331	340	445.582437	384	493.393483	428	537.468466
298.15	397.150308	342	447.830472	386	495.480491	430	539.379124
300	399.341102	344	450.071792	388	497.559682	432	541.281665
302	401.705009	346	452.306325	390	499.631025	434	543.176089
304	404.064103	348	454.534003	392	501.694495	436	545.0624
306	406.418266	350	456.754758	394	503.750066	438	546.940599
308	408.767384	352	458.968525	396	505.797712	440	548.810691
310	411.111344	354	461.175243	398	507.837412	442	550.672682
312	413.450036	356	463.374849	400	509.869145	444	552.526576
314	415.783352	358	465.567286	402	511.892891	446	554.372383
316	418.111187	360	467.752497	404	513.908632	448	556.210108
318	420.433437	362	469.930427	406	515.916352	450	558.039762
320	422.750001	364	472.101024	408	517.916034	452	559.861354
322	425.060782	366	474.264238	410	519.907666	454	561.674895
324	427.365683	368	476.420019	412	521.891236	456	563.480396
326	429.66461	370	478.568321	414	523.866731	458	565.277869
328	431.957471	372	480.709099	416	525.834142	460	567.067328
330	434.244179	374	482.84231	418	527.793462	462	568.848787
332	436.524645	376	484.967912	420	529.744681	464	570.62226
334	438.798785	378	487.085868	422	531.687796	466	572.387762
336	441.066516	380	489.196138	424	533.6228	468	574.14531
338	443.32776	382	491.298688	426	535.549691	470	575.89492

Table S5

Values calculated for *C*p of PA at different temperatures

	$C_{p,m}$ (g)		$C_{p,m}$ (g)		$C_{p,m}$ (g)
T/K	J mol^{-1} K^{-1}	T/K	J mol^{-1} K^{-1}	T/K	J mol^{-1} K^{-1}
296	132.923666	340	150.471286	384	166.854841
298.15	133.803232	342	151.243276	386	167.56835
300	134.558505	344	152.012798	388	168.279053
302	135.373344	346	152.77983	390	168.986943
304	136.186402	348	153.544349	392	169.692014
306	136.997634	350	154.306332	394	170.39426
308	137.806995	352	155.065759	396	171.093674
310	138.614444	354	155.822609	398	171.790251
312	139.419939	356	156.576864	400	172.483987
314	140.22344	358	157.328504	402	173.174877
316	141.024908	360	158.077511	404	173.862919
318	141.824306	362	158.823869		
320	142.621596	364	159.567562		
322	143.416744	366	160.308574		
324	144.209714	368	161.04689		

326	145.000473	370	161.782496
328	145.788989	372	162.51538
330	146.57523	374	163.245528
332	147.359166	376	163.972928
334	148.140768	378	164.69757
336	148.920006	380	165.419443
338	149.696855	382	166.138536

Table S6

Electronic energies for the different conformers of the 3,4- and 3,5-DABA and DIAs in E_h.

Conformer	Energy	Conformer	Eneregy
	3,4-DABA		
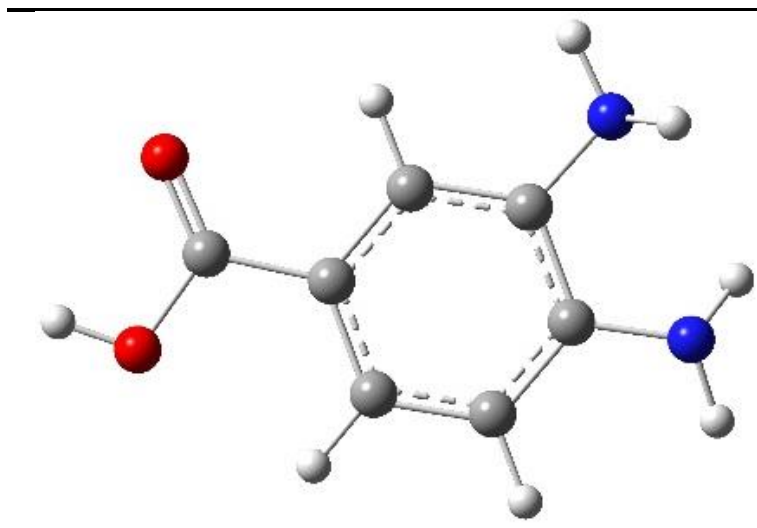	-531.704237	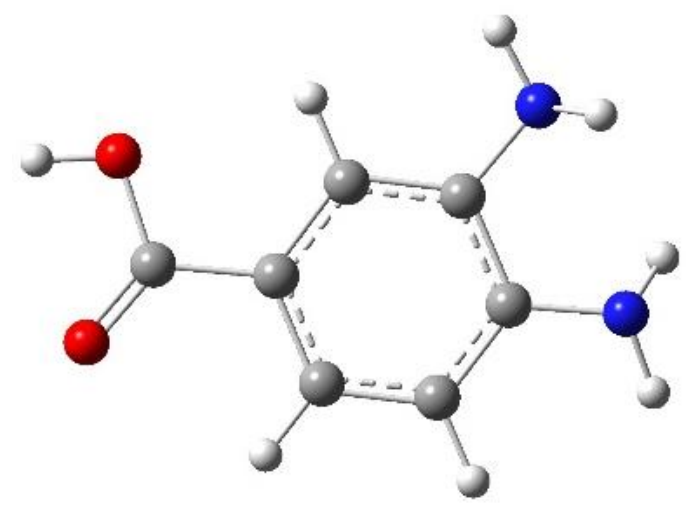	-531.704603
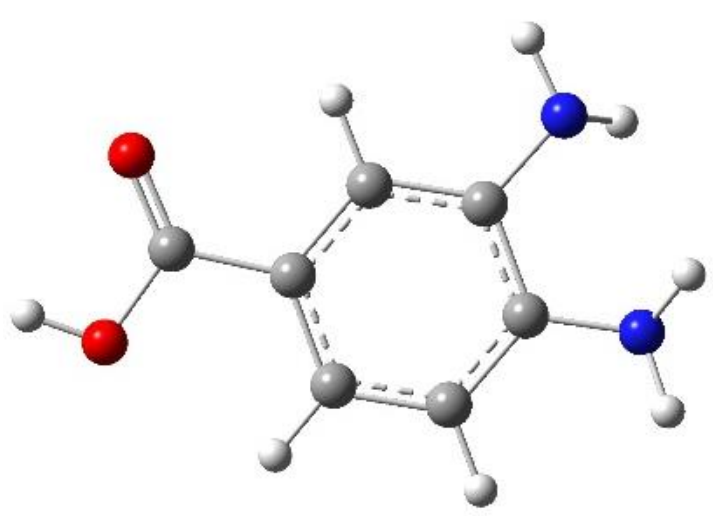	-531.70460	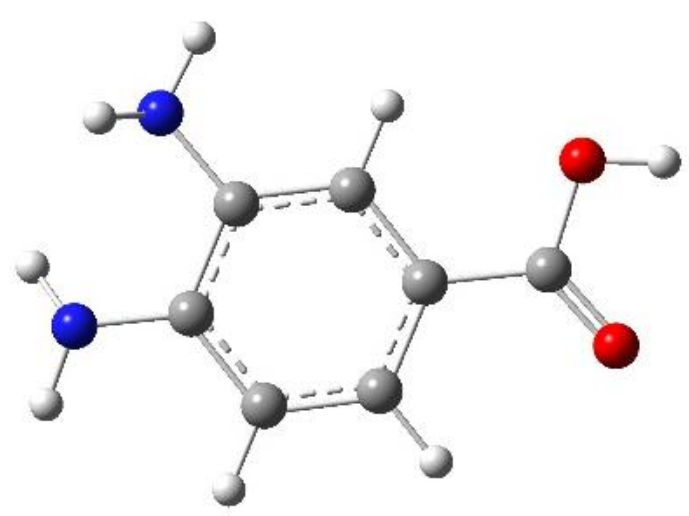	-531.704237
	3,5-DABA		
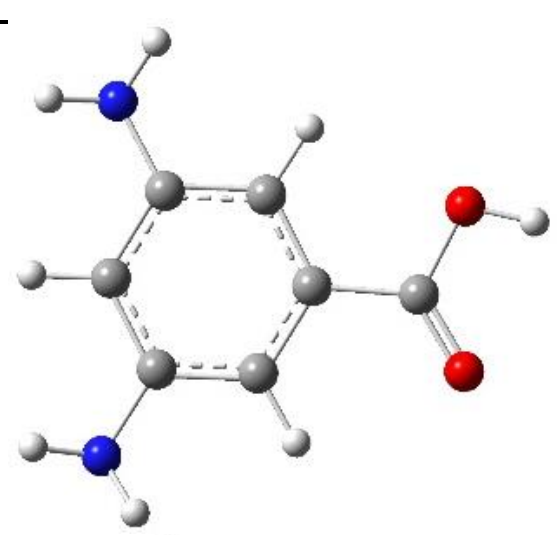	-531.701875	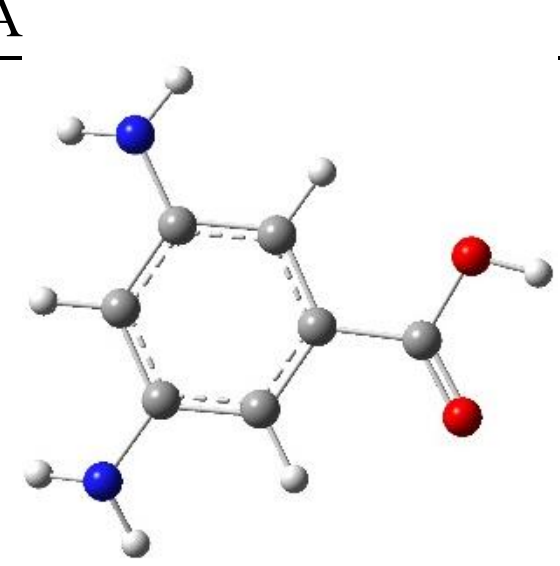	-531.701731
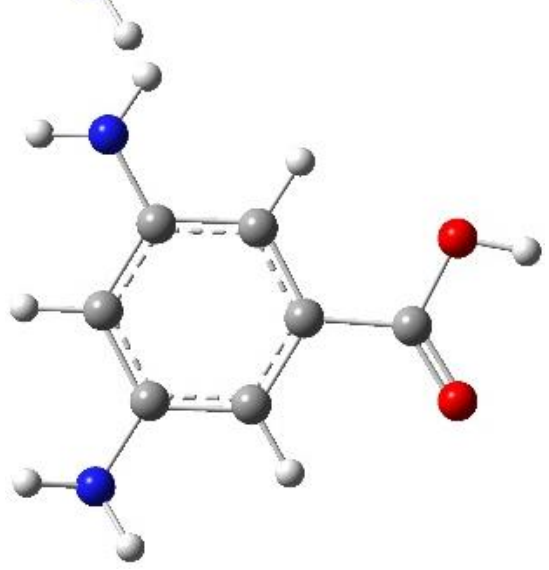	-531.701734	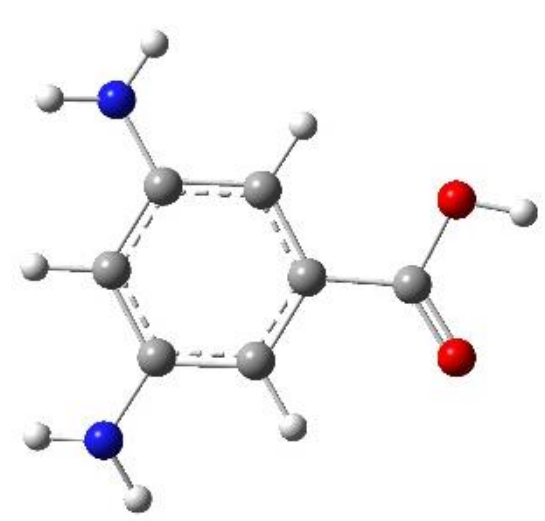	-531.701734
	3,4-DIA		

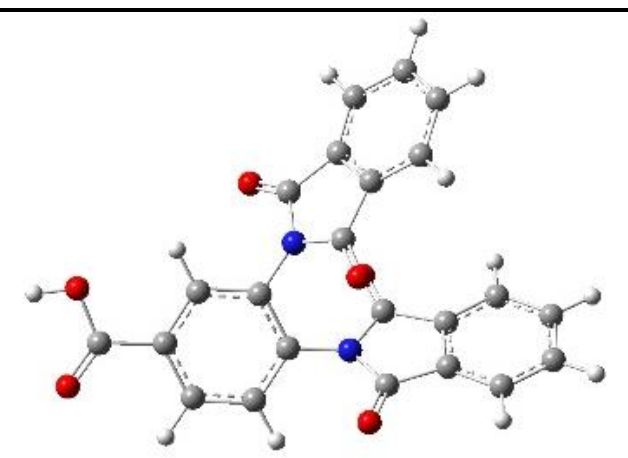	-1444.995125	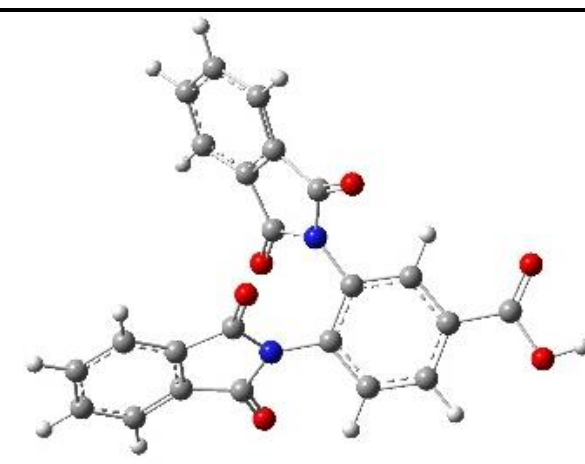	-1444.994983
3,5-DIA			
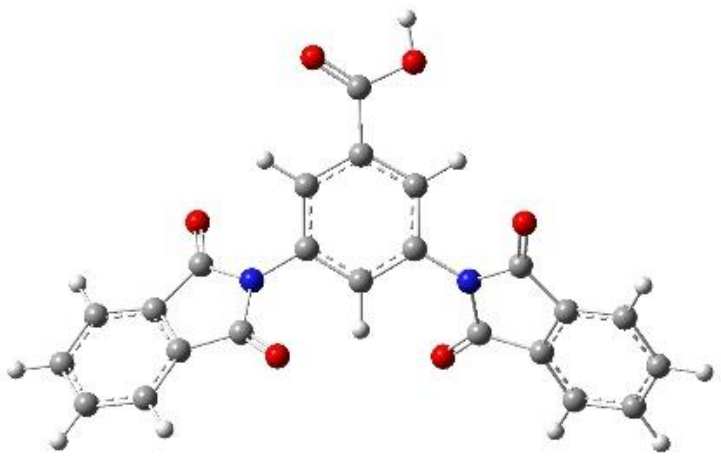	-1444.998551		
